# Bio-Inspired Artificial Intelligence Multimorbidity Score for Human–Machine Clinical Risk Stratification

**DOI:** 10.34133/cbsystems.0691

**Published:** 2026-09-30

**Authors:** Jinghao Liang, Ying Liu, Wei Wang, Zixian Xie, Yijian Lin, Jihao Qi, Yiwen Cai, Yongxuan He, Yaowen Liang, Yiling Chen, Suzheng Zhao, Ziqiu Cheng, Fayuan Wu, Haonan Zhao, Xinhao Liang, Kaiqi Zhang, Yuanlin Yuan, Jingchun Ni, Xiaoyao Lei, Liangyi Yao, Yuanqin Liu, Zishan Huang, Dianhan Lin, Weiqiang Yin, Hengrui Liang, Zhihua Guo, Qiu Wei, Feiying He, Mingyang Jiang, Nanshan Zhong, Qiong Liang, Jianxing He

**Affiliations:** ^1^ Department of Thoracic Surgery and Oncology, The First Affiliated Hospital of Guangzhou Medical University, China State Key Laboratory of Respiratory Disease and National Clinical Research Center for Respiratory Disease, Guangzhou 510120, China.; ^2^ Health Management Center, Yuebei People’s Hospital Affiliated to Shantou University Medical College, Shaoguan, Guangdong 512025, China.; ^3^ Second Clinical Medical College, Guangdong Medical University, Dongguan 523000, China.; ^4^ Department of Thoracic Surgery, The First Affiliated Hospital of Guangzhou Medical University, Guangzhou 510120, China.; ^5^ First Clinical College, Guangzhou Medical University, Guangzhou 511436, China.; ^6^ Shantou University Medical College, Shantou 515063, China.; ^7^ Department of Respiratory Medicine, Wuming Hospital of Guangxi Medical University, Nanning 530199, China.; ^8^ School of Health Management, Southern Medical University, Guangzhou 510515, China.; ^9^ Department of Respiratory Medicine, The First Affiliated Hospital of Guangzhou Medical University, China State Key Laboratory of Respiratory Disease and National Clinical Research Center for Respiratory Disease, Guangzhou Institute of Respiratory Disease, Guangzhou 510120, China.; ^10^ Department of Respiratory and Critical Care Medicine, The First People’s Hospital of Nanning, Nanning 530022, China.

## Abstract

Multimorbidity is strongly associated with mortality, but existing comorbidity indices often generalize poorly across populations because they depend on fixed disease lists and context-specific weights. We developed the large-language-model-based multimorbidity score (LLM-MMS), a prompt-based large-language-model-derived multimorbidity phenotype score, as a bio-inspired artificial intelligence score for human–machine clinical risk stratification and evaluated it for all-cause mortality prediction in 770,439 adults from 9 international longitudinal cohorts. Harmonized participant-level data were converted into standardized health narratives and processed without model fine-tuning. In the UK Biobank, LLM-MMS achieved strong discrimination, calibration, and prediction accuracy. Across 8 external cohorts, it maintained consistent performance, with *C*-indices of 0.67 to 0.86 and observed-to-expected ratios close to 1.00. This outperformed recalibrated conventional comorbidity indices in every cohort with absolute *C*-index gains of 0.12 to 0.21. Performance remained stable across sex and age subgroups, including adults younger than 60 years, and decision-curve analysis showed greater or comparable clinical net benefit in most cohorts. Proteomic and interpretability analyses supported the biological plausibility of LLM-MMS, linking high multimorbidity burden to inflammatory, metabolic, and tissue-damage pathways and identifying clinically coherent drivers of risk. These findings suggest that LLM-MMS offers a transportable and interpretable bio-inspired framework that integrates clinician-like multimodal reasoning with population-level validation for multimorbidity risk stratification across diverse populations.

## Introduction

Against the backdrop of global population aging and the growing burden of chronic diseases, multimorbidity has evolved from a characteristic of a small number of complex cases to a common condition that influences clinical decision-making, healthcare resource allocation, and research design [1–3]. A landmark Scottish primary-care study involving more than 1.75 million people reported that approximately 23% of the population had 2 or more chronic conditions; although multimorbidity increased steeply with age, the absolute number of affected individuals was greater among those younger than 65 years, with a clear socioeconomic gradient and evidence of earlier onset “aging forward” [1]. This heterogeneity stems from the differences in disease profiles, baseline risk, and healthcare systems, making risk stratification and comparability across countries and cohorts a shared methodological and practical challenge [2,3].

To support large-scale risk adjustment and prognostic assessment, clinical and epidemiological research has long relied on structured comorbidity or multimorbidity indices, including the Charlson comorbidity index (CCI) and its updated coding algorithms (Quan adaptation) [4,5], the Elixhauser comorbidity system and weighted summary scores (van Walraven score) [6,7], the functional comorbidity index (FCI) focused on functional outcomes [8], the Hospital Frailty Risk Score (HFRS) derived from inpatient electronic records [9], and International Classification of Diseases-coded multimorbidity-weighted indices (MWI) [10]. These tools are easy to implement and interpret, and can be incorporated into regression models or used for risk adjustment in payment and quality assessments; accordingly, they have been widely applied in multicohort research and real-world evidence studies [4–10].

However, multimorbidity measurement remains fragmented: There are substantial variations in disease lists, inclusion criteria, and weighting schemes, and no single gold standard has been established. A systematic review highlighted wide variation in disease coverage (from a handful to more than 100 conditions), the empirical basis for weighting, and intended use cases, limiting comparability across studies [11]. More importantly, the weights of conventional indices are typically derived from regression models fitted in specific countries, data sources, and outcome settings; when these indices are applied to populations with different health systems, coding practices, or case mix, they often exhibit calibration drift and reduced performance, requiring rigorous external validation and context-specific updates (recalibration or model revision) [12].

In recent years, transformer-based foundation models and large language models (LLMs) have offered a new approach to representing the complex structure of multimorbidity. Reviews of foundation models for generalist medical artificial intelligence (AI) have confirmed that these models can learn more transferable clinical representations through training on large-scale medical corpora and multimodal or multitask objectives, potentially serving as a general-purpose substrate across tasks and settings [13]. In electronic health records, generative pretrained transformers have been used to model patient timelines for prediction, underscoring the potential of sequential representations to capture disease co-occurrence and evolution [14]. Methodological discussions on clinical risk prediction with language models further argue that LLMs can flexibly integrate structured codes with clinical semantic information, potentially moving beyond fixed disease lists and fixed weights that constrain conventional indices [15,16]. At the same time, LLM deployment carries risks, including reliability fluctuations, cognitive or confirmation bias, and interaction-induced systematic errors that may be amplified across populations and tasks; therefore, robustness should be tested through multicohort external validation and stratified analyses [17,18].

From a bio-inspired and human–machine systems perspective, multimorbidity assessment can be viewed as an integrative state-estimation problem in a complex human biological system. Clinicians routinely synthesize distributed information across organ systems, physiological reserve, laboratory markers, functional status, lifestyle exposures, and social context to infer overall vulnerability and guide risk-oriented decisions. LLMs provide a computational substrate for approximating this form of integrative reasoning, not by replacing clinical judgment, but by transforming heterogeneous health signals into structured, reproducible, and auditable representations. In this study, we conceptualized a prompt-based large-language-model-based multimorbidity score (LLM-MMS) as a bio-inspired AI multimorbidity score that abstracts clinician-like cross-system reasoning into organ-specific biological ages, frailty age, and multidimensional health grades for human–machine clinical risk stratification.

Based on this underlying principle, we developed and validated LLM-MMS to summarize individual multimorbidity burden in a more expressive and portable manner for risk stratification. Across large longitudinal cohorts from multiple countries and regions, we benchmarked LLM-MMS against recalibrated conventional indices (CCI, Elixhauser, FCI, HFRS, and MWI) using harmonized external validation of discrimination (*C*-index), overall prediction error (Brier score), and calibration (observed-to-expected ratios). Additionally, we assessed stability and potential bias across key subgroups defined by sex and age (Fig. 1) [12,19].

**Fig. 1.**
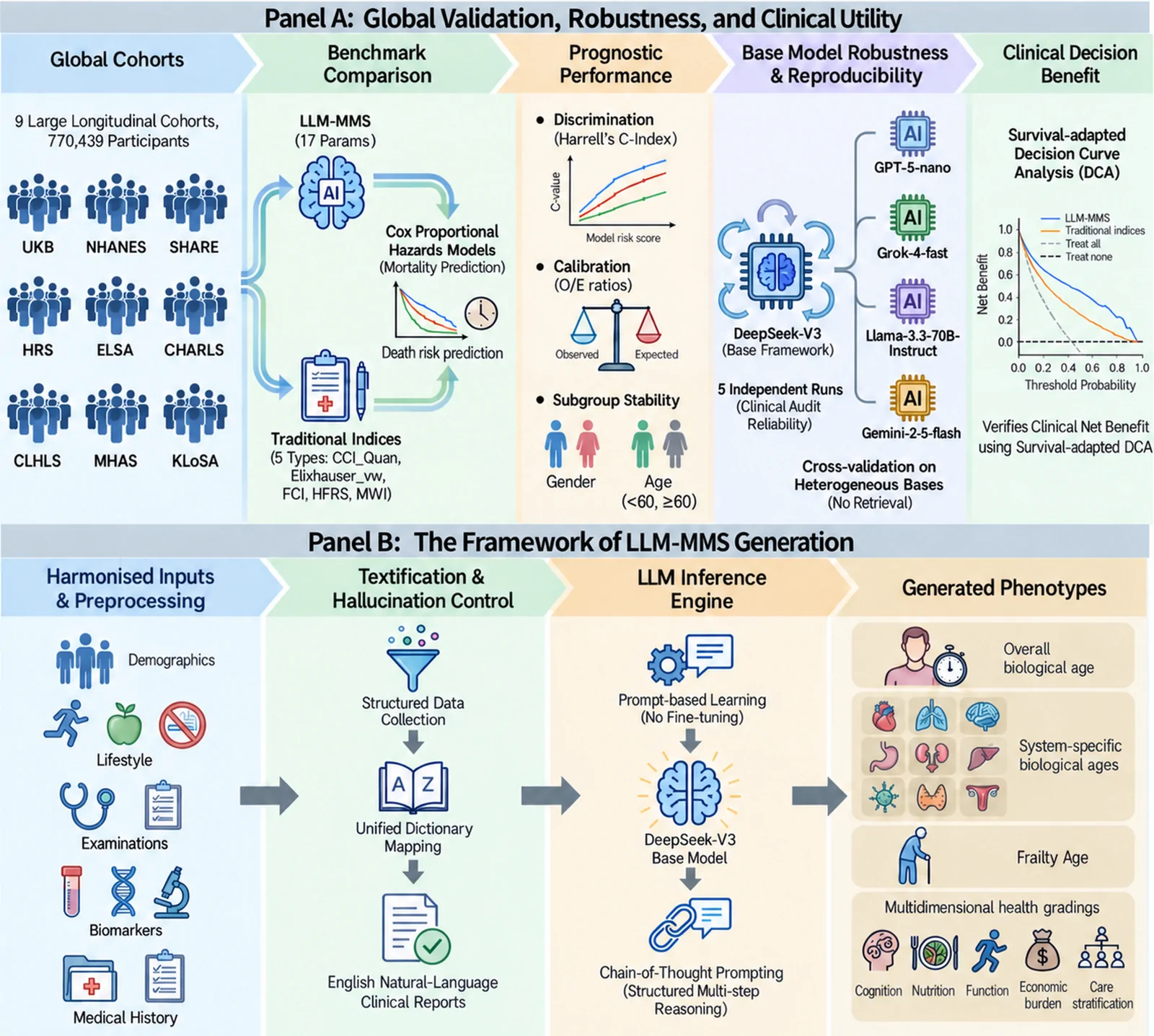
Overview of the LLM-MMS: global validation, robustness assessment, and generation framework. AI, artificial intelligence; CCI_Quan, Charlson comorbidity index (Quan version); CHARLS, China Health and Retirement Longitudinal Study; CLHLS, Chinese Longitudinal Healthy Longevity Survey; DCA, decision-curve analysis; ELSA, English Longitudinal Study of Ageing; Elixhauser_vw, Elixhauser comorbidity index (van Walraven version); FCI, functional comorbidity index; HFRS, Hospital Frailty Risk Score; HRS, Health and Retirement Study; KLoSA, Korean Longitudinal Study of Aging; LLM, large language model; LLM-MMS, large-language-model-based multimorbidity score; MHAS, Mexican Health and Aging Study; MWI, multimorbidity-weighted index; NHANES, National Health and Nutrition Examination Survey; O/E, observed-to-expected; SHARE, Survey of Health, Ageing and Retirement in Europe; UKB, UK Biobank. Panel A summarizes the global validation, robustness, and clinical utility evaluation of LLM-MMS across 9 large longitudinal cohorts (total *n* = 770,439). LLM-MMS (17 parameters) is benchmarked against 5 traditional comorbidity indices (CCI_Quan, Elixhauser_vw, FCI, HFRS, and MWI) using Cox proportional-hazards models for mortality prediction. Prognostic performance is assessed by discrimination (Harrell’s *C*-index), calibration (O/E ratios), and stability across demographic subgroups (sex; age <60 y vs. ≥60 y). Robustness and reproducibility are evaluated via a DeepSeek-V3 base framework with 5 independent runs and cross-validation across heterogeneous LLM backbones (no retrieval). Clinical decision benefit is assessed using survival-adapted DCA. Panel B illustrates the LLM-MMS generation pipeline: harmonized structured inputs (demographics, lifestyle, examinations, biomarkers, and medical history) are converted into standardized English natural-language clinical reports through unified dictionary mapping with hallucination control, followed by prompt-based learning (no fine-tuning) and chain-of-thought prompting to generate phenotypes including overall biological age, system-specific biological ages, frailty age, and multidimensional health gradings.

### Research in context

#### Evidence before this study

Multimorbidity is a major driver of mortality risk, healthcare use, and prioritization of preventive care. Yet, comorbidity indices commonly used in practice and research were developed in specific settings and can be poorly calibrated when applied to populations with different age structures, ascertainment methods, and health systems. This may misclassify risk and undermine equitable targeting of interventions. It remains unclear whether LLM-derived multimorbidity measures can improve the stratification of modifiable risks across countries.

#### Added value of this study

Using a multinational dataset from 9 longitudinal population cohorts, we employed a standardized preprocessing and prompt-based approach (without fine-tuning) to derive the LLM-MMS from routinely collected indicators. Compared with recalibrated CCI, Elixhauser, FCI, HFRS, and MWI, LLM-MMS provided more consistent discrimination and calibration across external cohorts and maintained performance in sex and age subgroups—especially among people younger than 60 years, where conventional indices often performed poorly. Decision-curve analyses suggested that, across most cohorts, the net benefit was greater or comparable across clinically relevant risk thresholds.

#### Implications of all the available evidence

More transportable multimorbidity risk stratification can support earlier identification of high-risk individuals, improve targeting of follow-up and prevention, and reduce cross-system miscalibration that can disadvantage particular subgroups. LLM-MMS is a promising approach for international research and population health management; however, its clinical implementation requires prospective evaluation, clear governance of model version control, and routine audits of calibration drift and subgroup equity.

## Methods

### Ethics statement

All participants included in the cohorts had provided written informed consent, and participation was voluntary with the right to withdraw at any time. The studies received ethical approval from their respective institutional review boards (IRBs) or ethics committees as follows: the UK Biobank (UKB) was approved by the North West Multi-centre Research Ethics Committee, with data use governed by the relevant Material Transfer Agreement; the US National Health and Nutrition Examination Survey (NHANES) by the National Center for Health Statistics Research Ethics Review Board; and the US Health and Retirement Study (HRS) by the University of Michigan IRB. For the Chinese cohorts, the China Health and Retirement Longitudinal Study (CHARLS) received approval from the Peking University IRB (11015/11014) and the Chinese Longitudinal Healthy Longevity Survey (CLHLS) from the Peking University Biomedical Ethics Committee (13074). European and other international cohorts were approved by the London Multi-Centre Research Ethics Review Committee (ELSA), the Max-Planck Society and University of Mannheim Ethics Council (SHARE), the Korea Centers for Disease Control and Prevention IRB (KLoSA), and the ethics bodies at the University of Texas Medical Branch, the National Institute of Statistics and Geography, and Mexico’s Instituto Nacional de Salud Pública (MHAS). Publicly available datasets (such as KLoSA and MHAS) and restricted datasets (UKB) were utilized in strict accordance with their respective data-usage policies.

### Study design and participants

We conducted a multicohort validation using deidentified individual-level data from 9 population-based cohorts (UKB, NHANES, SHARE, HRS, CHARLS, CLHLS, ELSA, KLoSA, and MHAS) to build an international longitudinal dataset. Each cohort had local ethics/governance approval and consent at baseline and follow-up as required. UKB recruited ~502,000 adults aged 40 to 69 years (2006–2010) with follow-up via linked health records. NHANES is an ongoing nationally representative US survey (1999 onward) combining interviews and standardized examinations, linked to mortality. HRS (1992), ELSA (2002), SHARE (2004), KLoSA (2006), MHAS (2001), and CHARLS (2011) are nationally representative longitudinal aging studies with ~2-year follow-up. CLHLS (1998) tracks the oldest old across 22 Chinese provinces with ~2- to 3-year follow-up.

### Data harmonization and preprocessing

We harmonized variables using a unified dictionary, standardizing definitions, units, coding, and time anchoring across demographics, lifestyle, examinations, biomarkers, and medical history while retaining cohort provenance. Model inputs were converted from structured variables into English natural-language reports with standard units, omission of missing items to reduce hallucination risk, hierarchical domain grouping, and chronologically normalized medical history narratives (Table S1). For non-English cohorts, including CHARLS, KLoSA, and MHAS, no participant-level free-text clinical narratives were translated. Instead, cohort-specific structured responses, measurements, and disease-history variables were mapped to harmonized English labels and standard units through the unified dictionary before report generation.

### Eligibility criteria

Participants were included if multimorbidity was present at the index assessment and survival follow-up time/event status were valid. Multimorbidity was defined as ≥2 conditions from a prespecified harmonized list [20]. Conditions were derived from International Classification of Diseases, 10th revision, records when available; otherwise, cohort-specific sources (self-report, medications, examination/laboratory definitions, and administrative linkages) were mapped to harmonized definitions. To leverage repeat waves, we used a “risk-period” framework where later waves contributed additional baselines if multimorbidity was met and events were not double-counted [21]. Within this framework, eligible wave-specific assessments contributed nonoverlapping follow-up intervals: Each interval started at the wave-specific assessment date and ended at the next eligible wave, death, or censoring, whichever occurred first. Covariates were fixed at the interval start, and death was assigned only to the final interval containing the death date.

We built an LLM-driven joint global and organ-specific framework to derive aging-related measures from routine indicators. Outputs included organ-specific biological ages for cardiovascular, digestive, respiratory, endocrine/metabolic, neurological, hematological, musculoskeletal/motor, urinary, and immune systems [22], a frailty age [9,23], and multidimensional health gradings (psychological health [1,24], diet [25,26], behaviors/habits [27], medical history [1], income [1,28], and family history [29]), integrated into an overall biological age construct.

We used prompt-based learning (without fine-tuning) [30] with chain-of-thought prompting to promote structured multistep reasoning [31]. The prompt is provided in Table S2. A single unified template combined (a) clinician-expert role + aging background; (b) task/output specifications for overall and organ-specific ages; and (c) participant narratives from harmonized variables. The model generated both overall and organ-specific ages simultaneously, accompanied by reasoning that supported organ-specific attention while maintaining consistency [32].

### Model development

The core reasoning engine of LLM-MMS employs DeepSeek-V3-0324. This model is a 671-billion-parameter Mixture-of-Experts (MoE) decoder-only Transformer, in which approximately 37 billion parameters are activated per token. Its core architecture includes Multi-head Latent Attention, the DeepSeekMoE module, and an auxiliary-loss-free load balancing mechanism (Supplementary Appendix, Section 1.4). Model inference is deployed on a high-performance computing cluster consisting of 8 NVIDIA A100 (80 GB) graphics processing units, with weights in FP8 E4M3 format. Weight-only FP8 quantization is implemented on the Ampere architecture, while activations use a BF16/FP16 mixed-precision representation to maintain stable inference quality while controlling memory overhead (Supplementary Appendix, Section 1.4).

At the input stage, LLM-MMS first maps standardized structured health variables into English natural-language health narrative reports, which are then provided to a unified 4-layer prompt template (Supplementary Appendix, Sections 1.3 and 1.6). This prompt template consists of a role-definition layer, a medical-knowledge-prior layer, a typed chain-of-thought constraint layer, and an output-contract layer based on JavaScript Object Notation. Through contextual constraints, it enables reasoning control oriented toward systems medicine and multimorbidity interactions (Supplementary Appendix, Section 1.6). The multimorbidity modeling task is further formalized as an 18-node directed acyclic graph, in which subtasks are sequentially unfolded according to topological dependencies within a single prompt call, rather than relying on multi-agent collaboration, recursive calls, or multiturn interactive reasoning frameworks (Supplementary Appendix, Section 1.5). This single-call, deterministic task-orchestration design reduces the complexity of the inference pathway and helps improve computational scalability, output reproducibility, and clinical auditability in large-scale population inference (Supplementary Appendix, Sections 1.2, 1.5, and 1.7).

During inference, LLM-MMS adopts a deterministic single-pass greedy decoding strategy, selecting at each generation step the token with the highest conditional probability as the output. It does not use stochastic test-time-scaling methods such as majority voting, best-of-N sampling, beam search, or Monte Carlo tree search (Supplementary Appendix, Section 1.7). This design constrains the model output to a deterministic result jointly determined by the fixed input, fixed prompt template, fixed backbone model, and fixed decoding rule, thereby ensuring that running the same participant record repeatedly under the same software and hardware configuration yields consistent multidimensional representations (Supplementary Appendix, Sections 1.7 and 1.10).

To test portability, we ran the same workflow (retrieval disabled) on GPT-5-nano (OpenAI) [33], Grok-4-fast (xAI) [34], Llama-3.3-70B-Instruct (Meta) [35,36], and Gemini-2.5-flash-preview-05-20 (Google) [36,37], evaluating cross-backend consistency in NHANES. We also repeated DeepSeek-V3 inference 5 times on NHANES to quantify output stability under identical inputs. The web-based survival prediction calculator used in this article is publicly available at http://llm-mms.xingyunjk.com/.

### Outcomes and predictors

The primary outcome was all-cause mortality. Follow-up was days from baseline (or wave-specific index date under the risk-period framework) to death or censoring. The predictor variables comprised LLM-derived measures of cardiovascular system age, digestive system age, respiratory system age, endocrine/metabolic system age, nervous system age, hematologic system age, musculoskeletal/motor system age, urinary system age, frailty age, immune system age, psychological health grading, dietary health grading, medical history, behavioral/habit health grading, income grading, family history grading, and overall biological age. Benchmarks included Quan’s Charlson index [5], FCI [38], HFRS [39], elixhauser_vw [7], and a multimorbidity-weighted index [40] using standard definitions.

### Missingness and omitted-information assessment

We evaluated whether missing data resulting from narrative structures based on omissions affected LLM-MMS outputs or prognostic performance [41]. First, cohort-specific missingness of key variables was summarized. Participant-level missingness, defined as the proportion of unavailable key variables, was then incorporated into Cox models per 10% increment. The primary LLM-MMS model was compared with models additionally adjusted for either overall missingness or variable-specific missingness indicators. Changes in hazard ratios, confidence intervals, *C*-score, and bootstrap-derived Δ*C*-score were evaluated.

To directly assess the effect of omitted information on LLM outputs, we conducted a paired sensitivity analysis in 5,000 participants by appending a standardized data-availability block to the original narratives, explicitly distinguishing observed, unavailable, and non-collected variables and prohibiting inference of missing values. Using the same LLM pipeline, agreement was assessed by Spearman correlation, intraclass correlation coefficient (ICC), Cox linear predictors, risk deciles, and top-decile reclassification.

### Biological and clinical applications of LLM-MMS

To explore biological correlates directly linked to LLM-MMS, we used the LLM-MMS-Cox linear predictor as a grouping variable and performed proteomic analysis on participants using available plasma proteomics data. Participants were ranked according to their LLM-MMS-derived mortality risk score, calculated from the 17-dimensional LLM-MMS phenotype using the Cox coefficients from the primary model. Differential protein abundance was then compared between the top 10% and bottom 10% of the LLM-MMS risk distribution. Age- and sex-adjusted linear models were used for differential abundance analysis, and *P* values were corrected for multiple testing using the Benjamini–Hochberg procedure. Ranked protein-level log2 fold changes from the top-versus-bottom decile comparison were further used for pathway enrichment analysis. We then evaluated the prognostic relevance of candidate proteins for all-cause mortality by fitting Cox proportional-hazards models in the proteomics subset and calculating the concordance index (*C*-index) with 95% confidence intervals (CIs) for each protein.

### Clinical interpretability of LLM-MMS

Clinical interpretability was assessed using structured model-generated rationales and systematic input-masking analyses [32]. For each inference, LLM-MMS produced 17 predefined outputs and corresponding rationales, which were processed by text normalization and keyword extraction to identify recurring clinical concepts.

Input-masking analyses were conducted for 9 prespecified risk factors: insufficient moderate-to-vigorous physical activity, elevated systolic blood pressure, elevated inflammatory markers, elevated pulse rate, alcohol consumption, current smoking, elevated glycated hemoglobin (HbA1c), body mass index (BMI) ≥28 kg/m^2^, and kidney dysfunction [42]. For each factor, 5,000 eligible participants were sampled, and the relevant input fields were replaced with “missing” while all other inputs, prompts, model settings, and output schemas were unchanged [43]. Original and masked outputs were passed through fixed Cox coefficients to calculate mortality risk scores. The masking effect was defined as the paired change in the Cox linear predictor and assessed using bootstrap 95% CIs and Wilcoxon signed-rank tests. Finally, masking-derived risk-factor importance rankings were compared with those based on attributable disability-adjusted life years from the Global Burden of Disease Study 2021 [42], and concordance was assessed using Spearman correlation.

### Statistical analysis

We used Cox proportional-hazards models to relate LLM-derived measures to mortality and compare prognostic performance versus traditional indices. To further address whether the added value of LLM-MMS was attributable to the LLM-derived representation rather than simply to the use of richer input variables, we additionally fitted raw-variable machine-learning survival models in the UKB, including random survival forest (RSF), XGBoost survival, and elastic-net Cox survival models, using the available structured variables directly [44–46]. Reporting was aligned with the Transparent Reporting of a Multivariable Prediction Model for Individual Prognosis or Diagnosis statement, with internal validation in UKB via 200 bootstrap resamples for optimism-corrected metrics [47–50]. The remaining 8 cohorts were used for external validation, with Level 2 recalibration for baseline-risk heterogeneity [51]. Discrimination used optimism-corrected Harrell’s *C*-index [49]. Ten-year calibration used plots, slope, and observed-to-expected (O/E) ratio; 10-year Brier scores used inverse probability of censoring weights [52]. Ten-year decision-curve analysis used survival-adapted net benefit with censoring adjustment [53,54]. For standard single-record analysis, variance and 95% CIs were estimated using a prespecified asymptotic method [55]. For Cox-type models based on repeated risk-period records, within-participant dependence was addressed using participant-clustered robust sandwich standard errors [56,57]. As a sensitivity analysis, we also fitted an extended Cox model using a counting-process start–stop formulation with nonoverlapping intervals [56,58]. Sampling was performed using a 2-stage stratified random sampling procedure based on vital status, follow-up-time tertiles within event and censored groups, sex, and baseline age quartiles [59]. Analyses were carried out using R 4.3.3 and Python 3.10.8; 2-sided *P* <0.05 defined significance.

## Results

### Study population and baseline characteristics

We integrated 9 independent cohorts (UKB, NHANES, SHARE, HRS, CHARLS, CLHLS, ELSA, MHAS, and KLoSA), comprising 770,439 participants from the UK, USA, Europe, China, Mexico, and South Korea (Fig. 2), with baseline assessments conducted between 1998 and 2018. Cohorts differed substantially in demographic and clinical profiles: Median age ranged from 47 years (NHANES) to 85 years (CLHLS), and women accounted for 52.0% to 65.3%. Key risk factors varied widely, including current smoking (7.9% in UKB to 39.5% in CHARLS), hypertension (32.2% in UKB to 86.4% in KLoSA), and diabetes (5.7% in UKB to 52.7% in NHANES). Multimorbidity distributions also differed: Most participants in KLoSA (100.0%), CHARLS (95.3%), and ELSA (95.0%) had 2 to 5 comorbidities, whereas a higher proportion had ≥10 comorbidities in NHANES (50.1%) and UKB (42.3%). Median follow-up ranged from 2.08 years (CHARLS) to 15.67 years (UKB), and all-cause mortality varied from 2.4% (ELSA) to 66.8% (CLHLS) (Table 1).

**Fig. 2.**
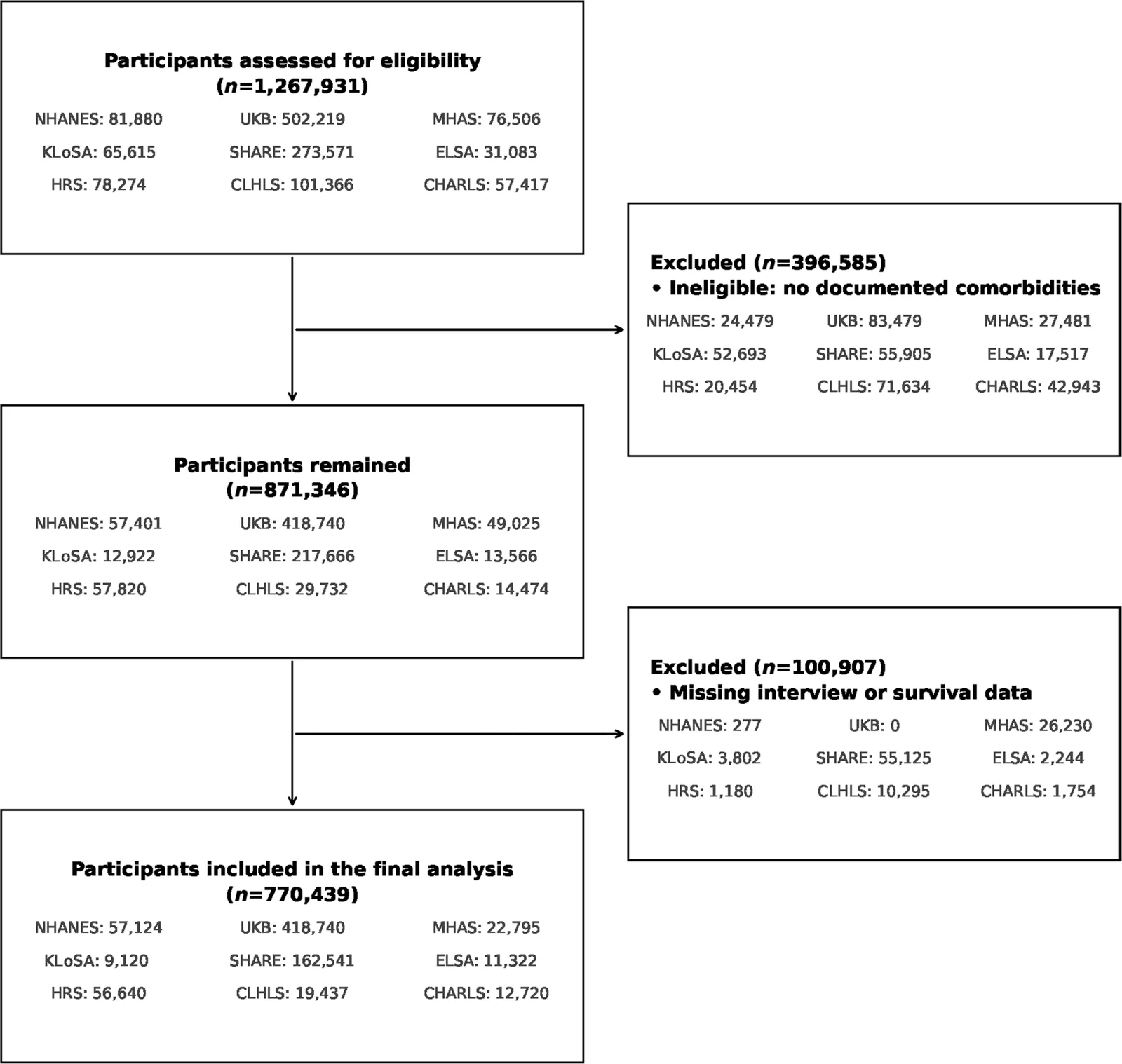
Flowchart of study participant selection across nine independent international cohorts. *n* represents the number of participants. A total of 1,267,931 individuals from 9 international cohorts were initially assessed for eligibility, of whom 770,439 were included in the final analysis after excluding individuals with missing data or those who did not meet the inclusion criteria.

**Table 1.** Baseline characteristics of study participants in 9 independent cohorts. Data are presented as number (percentage) for categorical variables and median (interquartile range, IQR) for continuous variables, unless otherwise indicated. Percentages may not total 100 because of rounding or missing data.

Characteristics	No./total/%								
	UKB (*n* = 418,740)	NHANES (*n* = 57,124)	SHARE (*n* = 162,541)	HRS (*n* = 56,640)	CHARLS (*n* = 12,720)	CLHLS (*n* = 19,437)	ELSA (*n* = 11,322)	MHAS (*n* = 22,795)	KLoSA (*n* = 9,120)
Country/region	UK	USA	Europe	USA	China	China	UK (England)	Mexico	Korea
Calendar year for examinations	2006–2010	1999–2018	2004–2017	2006–2012	2011–2015	1998–2014	2004–2013	2001–2015	2006–2018
Age, median (IQR), y	58 (51–64)	47 (31–64)	69 (58–73)	61 (54–68)	61 (54–69)	85 (75–93)	61 (55–68)	63 (62–76)	72 (63–75)
Sex									
Female	232,019 (55.4)	29,681 (52.0)	94,015 (57.8)	34,543 (61.0)	6,984 (54.9)	10,978 (56.5)	6,738 (59.5)	4,876 (65.3)	5,856 (64.2)
Male	186,721 (44.6)	27,443 (48.0)	67,684 (41.6)	22,096 (39.0)	5,736 (45.1)	8,459 (43.5)	4,584 (40.5)	7,908 (34.7)	3,264 (35.8)
Missing			842 (0.5)	1 (<0.1)				11 (<0.1)	
Medical history									
Current smoker	33,068 (7.9)	11,980 (21.0)	16,359 (10.1)	7,828 (13.8)	5,023 (39.5)	3,037 (15.6)	1,494 (13.2)	2,900 (12.7)	910 (10.0)
Diabetes	23,973 (5.7)	30,086 (52.7)	29,102 (17.9)	16,369 (28.9)	1,811 (14.2)	2,793 (14.4)	5,935 (52.4)	5,644 (24.8)	1,615 (17.7)
Hypertension	134,838 (32.2)	23,189 (45.6)	102,179 (62.9)	40,378 (71.3)	6,181 (48.6)	9,446 (48.6)	5,256 (46.4)	12,204 (53.5)	7,881 (86.4)
Cancer history	72,386 (17.3)	6,089 (10.7)	25,521 (15.7)	2,555 (4.5)	502 (3.9)	5,580 (28.7)	840 (7.4)	1,012 (4.4)	1,655 (18.1)
2–5 Comorbidities	140,412 (33.5)	5,660 (9.9)	134,322 (82.6)	44,630 (78.8)	12,126 (95.3)	12,078 (62.1)	10,752 (95.0)	19,255 (84.5)	9,117 (100.0)
6–9 Comorbidities	101,089 (24.2)	22,846 (40.0)	25,321 (15.6)	11,039 (19.5)	579 (4.6)	4,988 (25.7)	557 (4.9)	3,148 (13.8)	3 (<0.1)
10–15 Comorbidities	87,949 (21.0)	22,931 (40.1)	2,838 (1.7)	957 (1.7)	15 (0.1)	971 (5.0)	13 (0.1)	388 (1.7)	0
≥16 Comorbidities	89,290 (21.3)	5,687 (10.0)	60 (<0.1)	14 (<0.1)	0	1,400 (7.2)	0	4 (<0.1)	0
Self-reported general health state ^a^									
1 (Excellent/Very good)	52,594 (13.6)	6,331 (11.1)	22,538 (13.9) ^b^	3,408 (6.0)	212 (2.3)	1,193 (6.7)	799 (7.1)	161 (0.7)	27 (0.3)
2 (Very good/Good)	0	15,071 (26.4)		14,129 (25.0)	513 (5.6)	5,087 (28.7)	2,642 (23.4)	367 (1.7)	194 (2.1)
3 (Good/So so)	220,071 (56.8)	21,987 (38.5)	139,880 (86.1) ^c^	18,773 (33.2)	2,089 (22.8)	6,971 (39.3)	3,702 (32.9)	2,103 (9.6)	1,552 (17.1)
4 (Fair/Bad)	92,914 (24.0)	11,342 (19.9)		14,015 (24.8)	3,917 (42.7)	4,023 (22.7)	2,889 (25.6)	12,133 (55.5)	3,077 (33.8)
5 (Poor/Very bad)	21,825 (5.6)	2,352 (4.1)		6,259 (11.1)	2,450 (26.7)	446 (2.5)	1,236 (11.0)	5,388 (24.6)	4,270 (46.9)
Follow-up, median (IQR), y	15.67 (14.60–16.17)	9.25 (4.92–14.17)	4.17 (2.58–6.58)	8.17 (5.33–10.25)	2.08 (2.00–9.00)	3.21 (1.73–5.81)	6.17 (5.50–9.58)	10.25 (3.92–15.42)	4.08 (2.08–7.75)
Death during follow-up	50,701 (12.1)	9,013 (15.8)	35,424 (21.8)	22,072 (39.0)	1,507 (11.8)	12,989 (66.8)	268 (2.4)	7,559 (33.2)	2,204 (24.2)

CHARLS, China Health and Retirement Longitudinal Study; CLHLS, Chinese Longitudinal Healthy Longevity Survey; ELSA, English Longitudinal Study of Ageing; HRS, Health and Retirement Study; IQR, interquartile range; KLoSA, Korean Longitudinal Study of Aging; MHAS, Mexican Health and Aging Study; NHANES, National Health and Nutrition Examination Survey; SHARE, Survey of Health, Ageing and Retirement in Europe; UKB, UK Biobank.

^a^ Assessment of self-reported health status varied across cohorts. (a) US-based model: NHANES, HRS, ELSA, CHARLS, and MHAS utilized a 5-point scale (Excellent, Very good, Good, Fair, and Poor); (b) WHO/modified model: CLHLS and KLoSA employed a 5-point scale with different semantic descriptors (e.g., Very good, Good, So so/Fair, Bad/Poor, and Very bad/Very poor); (c) UKB model: A 4-point scale (Excellent, Good, Fair, and Poor) was used, which lacks a “Very good” category.

^b^ Defined as self-reported health status of “Excellent” or “Very good” in the SHARE cohort.

^c^ Defined as self-reported health status of “less than Very good” (e.g., Good, Fair, and Poor) in the SHARE cohort.

### Predictive performance of conventional comorbidity indices

Using UKB for model development, 5 conventional indices (CCI, Elixhauser, FCI, HFRS, and MWI) showed good internal calibration (O/E 0.99 to 1.01; 95% CI, 0.98 to 1.02) across sex and age subgroups (Table S3). In external validation, calibration deteriorated markedly and inconsistently across cohorts: overestimation was observed in ELSA (O/E 0.50 to 0.66), whereas underestimation predominated in SHARE, CHARLS, MHAS, and KLoSA (e.g., SHARE O/E 8.08 to 8.79; CHARLS 6.36 to 8.77; KLoSA 6.93 to 7.95). The largest deviation was in CLHLS, where HFRS yielded an O/E of 16.68 (95% CI, 16.39 to 16.97). Calibration error was strongly age dependent: In NHANES, conventional indices overestimated risk in participants aged <60 years (CCI O/E 0.49) but underestimated risk in those aged ≥60 years (O/E 2.21); in HRS, risk was underestimated by ~3-fold in those aged <60 years, whereas calibration was closer in those aged ≥60 years (O/E 0.97 to 1.18). In MHAS, miscalibration was most pronounced among women aged <60 years (O/E 14.41 to 17.22). Overall, conventional comorbidity scores showed substantial and unstable transportability across international population-based cohorts, with the largest calibration errors in older and high-risk settings (Table S4).

### Predictive performance and external validation

In the derivation cohort from the UKB, the LLM-MMS demonstrated strong discrimination, with a *C*-index of 0.81 (95% CI, 0.81 to 0.81) (Fig. 3A and Table 2), substantially higher than that of the best-performing recalibrated conventional index (FCI: 0.61 [95% CI, 0.60 to 0.61]; Δ*C*-index, 0.20) (Table 2). Calibration was excellent, with an O/E ratio of 0.99 (95% CI, 0.98 to 1.00), and overall prediction error was low (Brier score, 4.41%), indicating accurate absolute risk estimation. Across 8 external validation cohorts, the LLM-MMS maintained consistent discrimination, with *C*-indices ranging from 0.67 (CLHLS) to 0.86 (NHANES) (Table 2). In every validation cohort, discrimination exceeded that of the strongest recalibrated conventional comparator, with absolute improvements (Δ*C*-index) ranging from 0.12 to 0.21; the largest improvement was observed in CHARLS (Δ*C*-index, 0.21), despite assessment at a 5-year follow-up horizon compared with 10-year follow-up in the remaining cohorts. Calibration remained acceptable overall, with O/E ratios close to 1.00 (range, 0.94 to 1.04), indicating no substantial systematic overestimation or underestimation of risk. Brier scores were uniformly lower for the LLM-MMS than for conventional indices in all cohorts, reflecting improved overall predictive accuracy. Together, these findings demonstrate that the LLM-MMS achieved robust discrimination, preserved calibration, and reproducible performance across multiple external populations, supporting its generalizability across diverse international settings (Table S5).

**Fig. 3.**
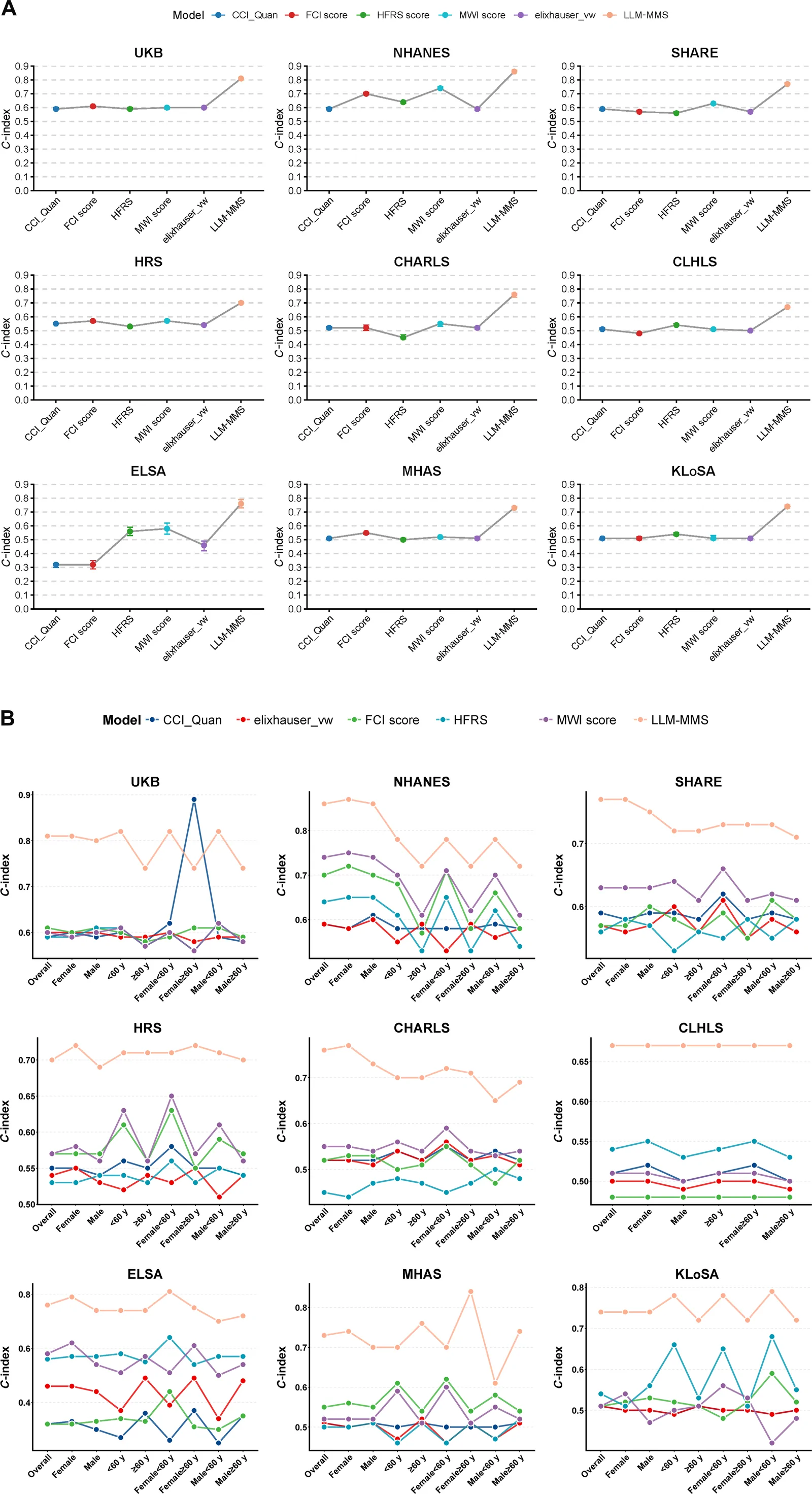
Comparison of predictive discrimination between LLM-MMS and five recalibrated traditional clinical indices across cohorts and demographic subgroups. Predictive performance is measured by the concordance index (*C*-index), with error bars representing 95% confidence intervals (CIs). The LLM-MMS is compared against 5 traditional comorbidity indices, all of which were recalibrated within each specific cohort using Cox proportional-hazards models to ensure a conservative and fair baseline comparison. Analysis was performed for the overall population and further stratified by sex (female, male) and age (<60 y and ≥60 y).

**Table 2.** Predictive performance of LLM-MMS versus the best-performing recalibrated conventional index in each cohort

Cohort	LLM-MMS			Best conventional index	Best index performance			Δ*C*-index (LLM—best)
	*C*-index (95% CI) ^a^	O/E (95% CI) ^b^	Brier /% ^c^		*C*-index (95% CI)	O/E (95% CI)	Brier /%	
UKB	0.81 (0.81 to 0.81)	0.99 (0.98 to 1.00)	4.41	FCI score	0.61 (0.60 to 0.61)	1.00 (0.99 to 1.01)	4.98	0.20
NHANES	0.86 (0.86 to 0.87)	0.96 (0.94 to 0.99)	8.54	MWI score	0.74 (0.74 to 0.75)	0.98 (0.96 to 1.00)	10.91	0.12
SHARE	0.77 (0.77 to 0.78)	1.04 (1.02 to 1.06)	17.61	MWI score	0.63 (0.63 to 0.63)	1.01 (1.00 to 1.02)	23.04	0.14
HRS	0.70 (0.70 to 0.71)	1.04 (1.01 to 1.07)	7.27	FCI score	0.57 (0.56 to 0.57)	1.00 (0.97 to 1.03)	7.49	0.13
CHARLS ^d^	0.76 (0.74 to 0.77)	0.94 (0.89 to 1.00)	10.46	MWI score	0.55 (0.53 to 0.56)	0.92 (0.87 to 0.98)	12.15	0.21
CLHLS	0.67 (0.66 to 0.67)	1.00 (0.99 to 1.02)	15.47	HFRS score	0.54 (0.54 to 0.55)	1.00 (0.98 to 1.02)	17.35	0.13
ELSA	0.76 (0.73 to 0.79)	0.98 (0.87 to 1.11)	2.61	MWI score	0.58 (0.54 to 0.62)	0.99 (0.88 to 1.12)	2.71	0.18
MHAS	0.73 (0.73 to 0.74)	0.96 (0.93 to 0.98)	15.40	FCI score	0.55 (0.54 to 0.55)	1.01 (0.99 to 1.04)	19.52	0.18
KLoSA	0.74 (0.73 to 0.75)	0.97 (0.93 to 1.01)	17.32	HFRS score	0.54 (0.53 to 0.55)	1.01 (0.96 to 1.05)	21.92	0.20

CHARLS, China Health and Retirement Longitudinal Study; CI, confidence interval; CLHLS, Chinese Longitudinal Healthy Longevity Survey; ELSA, English Longitudinal Study of Ageing; FCI, functional comorbidity index; HFRS, Hospital Frailty Risk Score; HRS, Health and Retirement Study; KLoSA, Korean Longitudinal Study of Aging; LLM-MMS, large-language-model-based multimorbidity score; MHAS, Mexican Health and Aging Study; MWI, multimorbidity-weighted index; NHANES, National Health and Nutrition Examination Survey; SHARE, Survey of Health, Ageing and Retirement in Europe; UKB, UK Biobank

^a^ The concordance index (*C*-index) represents the model’s discrimination ability, with 0.50 indicating no predictive power and 1.00 indicating perfect discrimination.

^b^ An observed/expected (O/E) ratio of 1.00 indicates perfect calibration; ratios >1.00 or <1.00 indicate underestimation or overestimation of risk, respectively.

^c^ Brier score measures the accuracy of probabilistic predictions. It represents the mean squared difference between the predicted probability and the actual outcome. A lower value indicates better predictive performance.

^d^ Special consideration for follow-up duration: For the CHARLS cohort, predictive performance was assessed at a 5-year follow-up node, whereas a 10-year follow-up node was utilized for all other 8 cohorts.

### Sensitivity analyses addressing missingness and repeated measurements

Across cohorts, the key-variable missingness ranged from 0% to 20% (Table S6). Adjustment for overall missingness did not materially affect model discrimination, with the *C*-index remaining unchanged before and after adjustment (0.80697 for both; paired Δ*C*-index, 0.0000004; bootstrap 95% CI, −0.0000004 to 0.0000012). Overall missingness was not associated with mortality (hazard ratio [HR] per 10% increase, 1.0007; 95% CI, 0.9893 to 1.0122; *P* = 0.904) (Table S7). Incorporating variable-level missingness indicators resulted in a modest improvement in discrimination (*C*-index, 0.80879; paired Δ*C*-index, 0.00182; bootstrap 95% CI, 0.00161 to 0.00203), suggesting that variable availability contained limited prognostic information (Table S8).

In the paired narrative sensitivity analysis, the outputs generated by explicit data availability narratives were broadly consistent with those based on omitted narratives. Across the 17 LLM-derived variables, the median ICC and Spearman correlation were 0.892 and 0.887, respectively. Cox linear predictors also showed high agreement between the 2 narrative formats (Spearman correlation, 0.896; ICC, 0.901), with only a small mean shift in the Cox linear predictor (Δ = 0.0186). Overall, 77.5% of participants remained within one risk decile, and 6.7% were reclassified into or out of the top-risk decile (Table S9).

In sensitivity analyses of external validation cohorts, after accounting for the correlations among repeated prediction records from the same participant, robust standard errors were only modestly larger than conventional standard errors, with increases ranging from 10.6% to 16.3% (Table S10). Model discrimination remained broadly consistent with the primary analyses, with *C*-indices ranging from 0.655 to 0.780 and a maximum absolute difference of 0.036 compared with the primary estimates. After recalibration, the absolute O/E bias for SHARE, CHARLS, CLHLS, and ELSA ranged from 0.028 to 0.066 relative to 1.00, while MHAS and KLoSA showed larger changes at 0.247 and 0.235, respectively. Brier scores improved in 5 of the 6 cohorts, with Δ Brier ranging from −7.81 to −0.70, and increased only in CLHLS by 6.46. Detailed results for the repeated-wave analyses are provided in Table S11.

### Subgroup analysis and robustness across demographics

In stratified analyses by sex and age, the LLM-MMS model retained stable discrimination, whereas conventional indicators showed marked variability (Fig. 3B). In NHANES, LLM-MMS *C*-statistics were 0.87 in females and 0.86 in males, compared with recalibrated conventional models clustering around 0.60 (Tables S4 and S5). Among participants aged <60 years—where conventional scores frequently exhibited floor effects—recalibrated CCI discrimination was particularly low (ELSA <60: 0.27; NHANES: 0.58), whereas LLM-MMS remained substantially higher (NHANES: 0.78; ELSA: 0.70; SHARE: 0.72). In participants aged ≥60 years, LLM-MMS also remained superior (SHARE: 0.72; MHAS: 0.76). However, differences between female and male subgroups were more evident in CHARLS and ELSA among participants aged <60 years and in MHAS across age strata. Notably, in the oldest-old male subgroup in CLHLS (≥60 y), conventional indicators showed limited discrimination (0.35 to 0.50), while LLM-MMS preserved a *C*-statistic of 0.67 (Table S5).

### Decision-curve analysis

Across the prespecified threshold-probability range of 0.01 to 0.35, the recalibrated pred_LLM demonstrated generally favorable decision-analytic performance across most external validation cohorts, with net benefit exceeding that of the default treat-all and treat-none strategies as well as the conventional predictors (pred_CCI, pred_ECI, pred_FCI, pred_HFRS, and pred_MWI) for both 5-year and 10-year mortality prediction. This pattern was less pronounced in ELSA and HRS. In ELSA, pred_LLM conferred only limited incremental net benefit at both prediction horizons, with the decision curves approaching the treat-none reference except at very low threshold probabilities. In HRS, decision-analytic utility was similarly attenuated; for 5-year prediction, pred_LLM and multiple conventional predictors yielded negative net benefit across part of the lower threshold-probability range (Fig. 4).

**Fig. 4.**
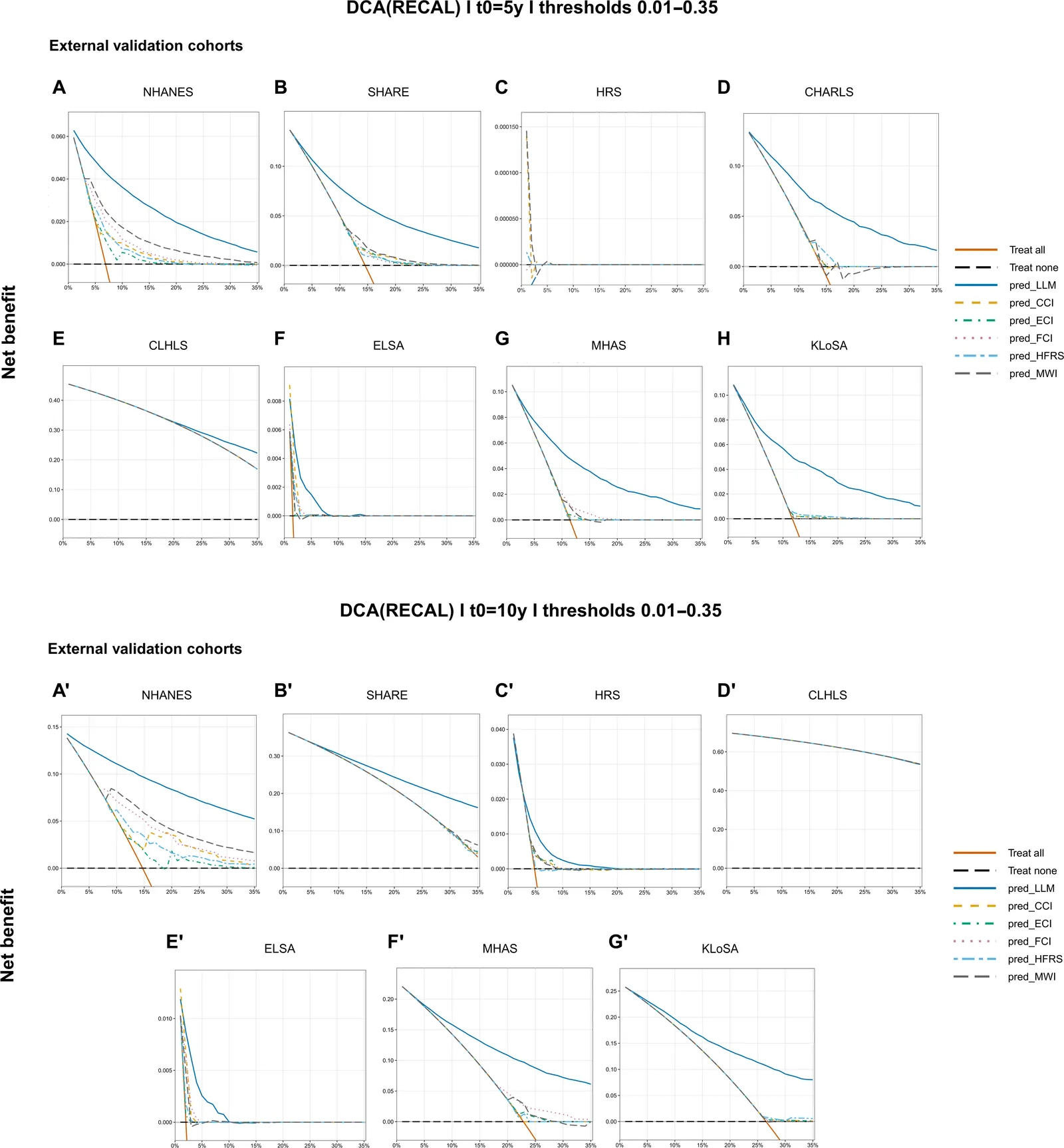
Survival-adapted DCA comparing clinical net benefit between LLM-MMS and 5 recalibrated traditional clinical indices for 5-year and 10-year mortality prediction across external validation cohorts. *t*_0_, prediction time horizon. Clinical decision benefit was evaluated using survival-adapted DCA across threshold probabilities of 0.01 to 0.35. Net benefit (*y*-axis) is plotted against threshold probability (*x*-axis), with “Treat all” and “Treat none” representing default strategies; higher net benefit indicates greater clinical utility. The *y*-axis was extended below zero to display threshold ranges in which a model or the treat-all strategy yielded negative net benefit. In DCA, the treat-none strategy corresponds to a net benefit of zero; values below zero indicate net harm relative to treating none. LLM-MMS (pred_LLM) is compared with 5 traditional indices (pred_CCI, pred_ECI, pred_FCI, pred_HFRS, and pred_MWI), all recalibrated within each cohort using Cox proportional-hazards models to provide a conservative and fair baseline comparison. The 5-year horizon panel (*t*_0_ = 5 y) includes 8 external cohorts (panels A to H: NHANES, SHARE, HRS, CHARLS, CLHLS, ELSA, MHAS, and KLoSA), while the 10-year horizon panel (*t*_0_ = 10 y) includes 7 external cohorts (panels A′ to G′: NHANES, SHARE, HRS, CLHLS, ELSA, MHAS, and KLoSA).

### LLM-MMS provided incremental discrimination beyond raw structured variables

We benchmarked survival models trained directly on the same raw structured variables in UKB as comparators to LLM-MMS. Among the raw-variable models, XGBoost survival showed the highest discrimination, with a *C*-index of 0.778, followed by elastic-net Cox regression (0.771) and random survival forest (0.725). LLM-MMS demonstrated superior discrimination, achieving a *C*-index of 0.810 (95% CI, 0.810 to 0.810). The time-dependent AUCs at 1, 3, 5, 10, and 15 years were 0.824, 0.800, 0.785, 0.784, and 0.795 for XGBoost survival; 0.808, 0.789, 0.775, 0.775, and 0.789 for elastic-net Cox regression; and 0.781, 0.753, 0.737, 0.735, and 0.740 for RSF, respectively.

### Portability across base LLMs and reproducibility

Since LLM-MMS depends on an external LLM at inference, we assessed its sensitivity to the choice of base LLM and to repeated runs. Among alternative base LLMs, discrimination varied only modestly with overlapping 95% CIs (area under the curve [AUC] across 1 to 18 years approximately 0.874 to 0.913; Uno’s *C* 0.864 to 0.889; Harrell’s *C* 0.866 to 0.878), supporting portability; grok-4-fast was highest or tied highest for most horizons, whereas llama-3.3-70b-instruct was slightly lower overall (Table S12). With the base LLM fixed, 5 independent repeated runs produced near-identical performance (maximum variation across horizons ≤0.004 for AUC, ≤0.004 for Uno’s *C*, and ≤0.002 for Harrell’s *C*), indicating high reproducibility (Table S13).

### Proteomic signatures associated with LLM-MMS risk

Participants with available plasma proteomics data were ranked according to the LLM-MMS-derived Cox linear predictor, and differential protein abundance was compared between the top 10% and bottom 10% of the mortality-risk distribution. Proteins with higher abundance in the top-risk decile included FGF21, ALPP, FABP1, GAST, OXT, LEP, IL6, HAVCR1, KRT18, and GDF15 (log2 fold change = 1.608 to 0.999) (Table S14). These proteins were consistent with biological processes involving inflammatory and cellular stress signaling, immune activation, epithelial/tubular injury, and metabolic/lipid stress responses (Fig. 5A). Gene-set enrichment analysis showed enrichment of pathways related to inflammatory regulation, antimicrobial humoral response, leukocyte chemotaxis, myeloid activation, fatty acid transport, and lipid oxidation (Fig. 5B). We then evaluated the prognostic value of the candidate proteins, and the results showed that they had varying degrees of discriminative ability for all-cause mortality. GDF15 performed best (*C*-score = 0.742; 95% CI, 0.736 to 0.746), followed by EDA2R (0.716; 95% CI, 0.703 to 0.725), TNFRSF10B (0.703; 95% CI, 0.691 to 0.715), WFDC2 (0.698; 95% CI, 0.686 to 0.715), and IGFBP4 (0.675; 95% CI, 0.665 to 0.689) (Fig. 5C). Based on this, we further constructed a protein-based prognostic model. Its AUCs at 1, 3, 5, 10, and 15 years were 0.872, 0.848, 0.828, 0.815, and 0.818, respectively, indicating that protein features had stable prognostic value across short-term and medium-to-long-term follow-up (Fig. 5D).

**Fig. 5.**
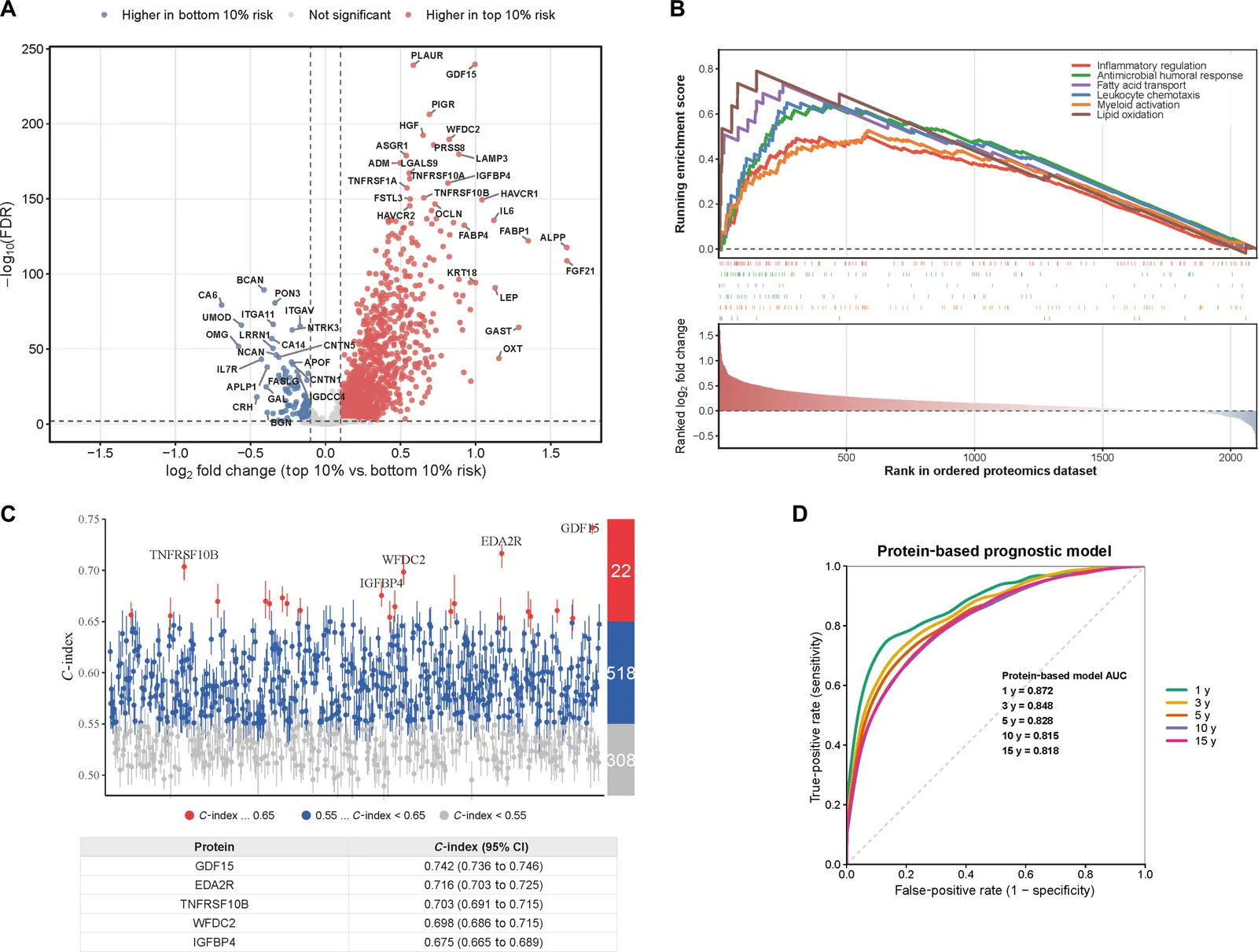
Proteomic signatures associated with LLM-MMS risk. (A) Volcano plot showing differential protein abundance between participants in the top 10% and bottom 10% of the LLM-MMS-derived mortality risk distribution. Proteins higher in the top 10% risk group are shown in red, whereas proteins higher in the bottom 10% risk group are shown in blue. Representative proteins with large effect sizes or high statistical significance are labeled. FDR, false discovery rate. (B) Gene-set enrichment profiles based on ranked log2 fold changes from the top-versus-bottom LLM-MMS risk comparison. Six enriched pathways are shown: inflammatory regulation, leukocyte chemotaxis, antimicrobial humoral response, myeloid activation, fatty acid transport, and lipid oxidation. (C) Prognostic performance of candidate proteins for all-cause mortality, assessed by *C*-index. Representative proteins with stronger discriminatory ability, including GDF15, EDA2R, TNFRSF10B, WFDC2, and IGFBP4, are highlighted, and their *C*-indices with 95% CIs are summarized in the table. (D) Time-dependent receiver operating characteristic curves of the XGBoost-Cox model constructed from selected protein features for predicting all-cause mortality at 1, 3, 5, 10, and 15 years. The model achieved areas under the curve (AUCs) of 0.872, 0.848, 0.828, 0.815, and 0.818, respectively, indicating stable short-term and medium-to-long-term prognostic performance.

### Interpretability analysis of LLM-MMS for multimorbidity risk assessment

We prompted the LLM to use chain-of-thought (CoT) reasoning, allowing representative intermediate reasoning steps for system-specific age and multidimensional health grading to be reviewed (Fig. 6A). Keyword analysis showed that physical activity, BMI, blood pressure, glucose, smoking, sleep, diet, medical history, family history, and cholesterol were frequently mentioned for overall biological age; respiratory age was mainly associated with spirometric function, cough, dyspnea, and smoking status, whereas cardiovascular and metabolic outputs were linked to blood pressure, lipids, HbA1c, glucose, and BMI (Fig. 6B), consistent with established clinical knowledge.

**Fig. 6.**
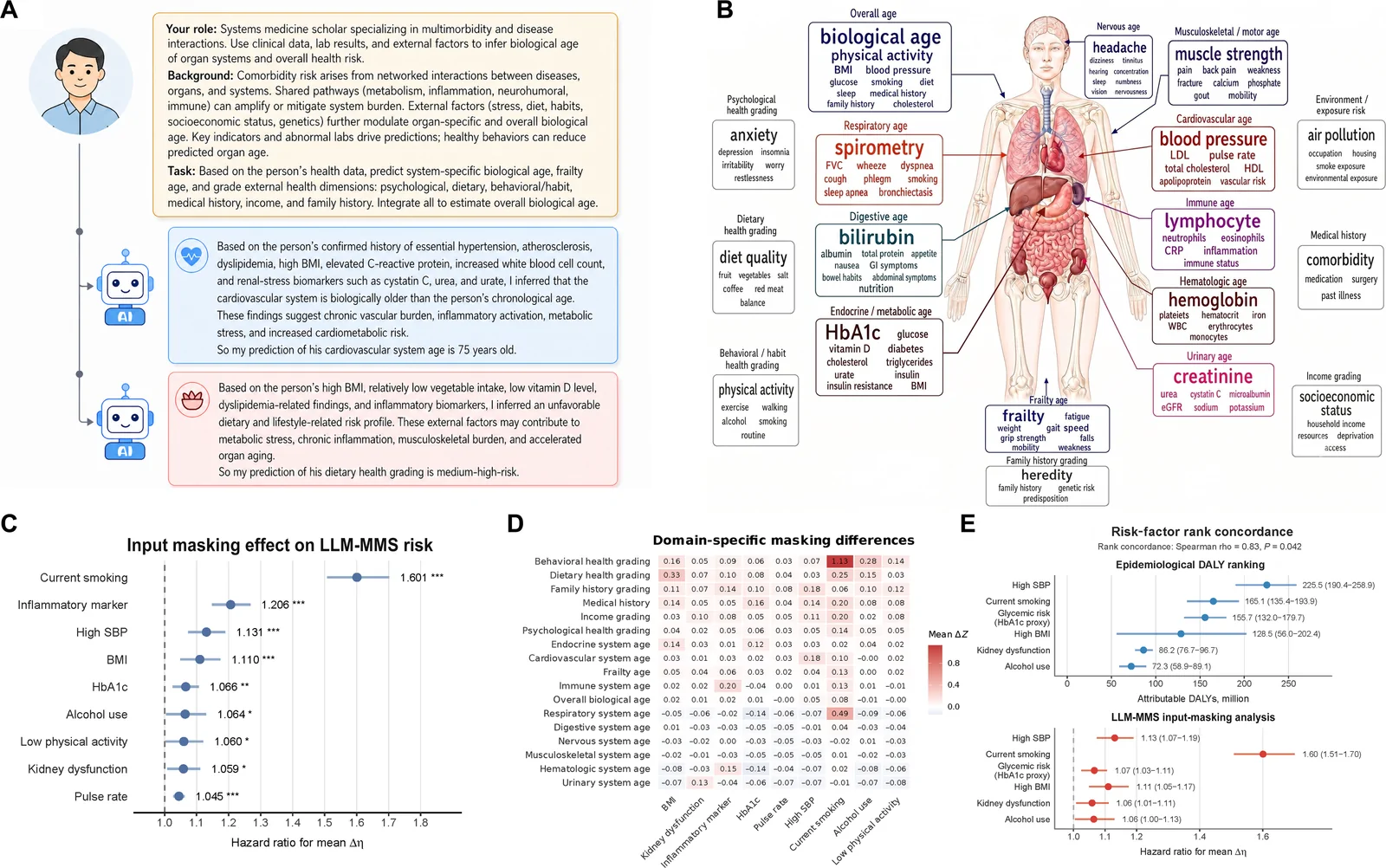
Interpretability and input-masking analyses of LLM-MMS. (A) Example of chain-of-thought reasoning generated by LLM-MMS, illustrating how the model integrates clinical characteristics, laboratory findings, and external health dimensions to infer organ-specific biological age and multidimensional health grading. (B) Keyword-based summary of model reasoning derived from chain-of-thought outputs. The word clouds highlight major determinants of overall biological age, organ-specific biological ages, frailty, and multidimensional health classifications, including psychological health, dietary health, behavioral/habit health, medical history, income, and family history. (C) Overall input-masking effects on the final LLM-MMS mortality risk score. Risk-factor-related input fields were replaced with “missing”, while all other report content, prompt, LLM backbone, decoding rule, and output schema were held fixed. Points show model-derived hazard ratios corresponding to the mean paired change in the Cox linear predictor; error bars indicate 95% CIs. Asterisks in (C) denote statistical significance: *P < 0.05, **P < 0.01, and ***P < 0.001. (D) Domain-specific masking differences across the 17 LLM-derived outputs. Cells show mean standardized paired changes between original and masked reports; positive values indicate higher output values under the original report than under the masked report. (E) Concordance between masking-derived risk-factor ranking and external epidemiological ranking based on attributable disability-adjusted life years from the Global Burden of Disease Study 2021. Rank concordance was assessed using Spearman rank correlation coefficient. AI, artificial intelligence; BMI, body mass index; HbA1c, glycated hemoglobin; FVC, forced vital capacity; GI, gastrointestinal; LDL, low-density lipoprotein; HDL, high-density lipoprotein; CRP, C-reactive protein; WBC, white blood cell; eGFR, estimated glomerular filtration rate; SBP, systolic blood pressure; DALY, disability-adjusted life year.

Input-masking analysis further showed that removing prespecified risk-factor information reduced the final LLM-MMS mortality risk score, with all 9 masking effects corresponding to HRs greater than 1.00 (range, 1.045 to 1.601; Fig. 6C). The largest effect was observed for current smoking, with a mean change in the Cox linear predictor of 0.471 and a model-derived HR of 1.60 (95% CI, 1.51 to 1.70; *P* = 5.42 × 10^−37^). Other notable effects were observed for inflammatory markers (HR, 1.21; 95% CI, 1.15 to 1.27), high systolic blood pressure (HR, 1.13; 95% CI, 1.07 to 1.19), BMI (HR, 1.11; 95% CI, 1.05 to 1.17), and HbA1c (HR, 1.07; 95% CI, 1.03 to 1.11) (Table S15).

Domain-specific masking heatmaps revealed corresponding output-level changes across the 17 LLM-derived phenotypes (Fig. 6D). Current smoking produced the largest domain-level change, mainly in behavioral health grading (mean Δ*Z* = 1.13) and respiratory system age (0.49). Inflammatory-marker masking mainly affected immune system age (0.20) and hematologic system age (0.15), whereas high systolic blood pressure affected cardiovascular system age (0.18). BMI masking was most prominent for dietary health grading (0.33), behavioral health grading (0.16), medical history (0.14), and endocrine system age (0.14), whereas HbA1c masking affected medical history (0.16) and endocrine system age (0.12). Kidney dysfunction showed its largest positive domain-level change in urinary system age (0.13) (Table S16).

Moreover, among risk factors with comparable Global Burden of Disease Study 2021 definitions, the masking-derived ranking was concordant with the external disability-adjusted-life-years-based epidemiological ranking (Spearman *ρ* = 0.83, *P* = 0.042; Fig. 6E). Together, these results indicate that LLM-MMS showed consistent interpretability evidence across CoT keyword summaries, input-masking perturbations, and external epidemiological comparison.

A web-based calculator implementing the final survival prediction model is available online at http://llm-mms.xingyunjk.com/.

## Discussion

Multimorbidity is becoming increasingly [1,60] important in health systems of aging societies and shows clear social patterning. Comorbidity indices such as the CCI [4], the Elixhauser measure [6], the FCI [8], the MWI [61], and claims-based frailty tools such as the HFRS [9] are widely used for risk adjustment and prognostication. However, most rely on fixed condition lists and fixed weights, and the meaning of a given diagnosis—and its associated risk—depends strongly on case mix, coding practices, baseline risk, and measurement context [62].

Across 9 large longitudinal cohorts (770,439 participants with baseline assessments spanning 1998–2018), traditional indices calibrated well in internal validation within the UK Biobank training environment, with O/E ratios close to 1.00 and broadly consistent across sex and age strata. In contrast, transport to 8 external cohorts produced large and inconsistent calibration drift, with directionally opposite errors across settings. For example, ELSA showed systematic overestimation (O/E ≈ 0.50 to 0.66), whereas SHARE, CHARLS, MHAS, and KLoSA showed pronounced underestimation (SHARE O/E ≈8.08 to 8.79; CHARLS ≈6.36 to 8.77). In CLHLS, HFRS reached an O/E ratio of 16.68. These findings underscore the context dependence of fixed-weight indices and echo prior concerns about their external validity [62].

Miscalibration also varied by age within cohorts, indicating that a single “relative risk scale” can warp as baseline risk, ascertainment, and case-mix change across the life course. In NHANES, participants younger than 60 years were often overestimated (CCI O/E 0.49), whereas those aged 60 years and older were underestimated (O/E 2.21); in HRS, the largest drift occurred among those younger than 60 years but attenuated in older adults. This pattern matters because the absolute number of people living with multimorbidity can be greater below age 65 years [1], and earlier onset is concentrated in socioeconomically deprived groups [1,60]. Tools that lose resolution or miscalibrate in younger adults risk missing opportunities for earlier prevention and follow-up.

Second-stage recalibration corrected average risk but rarely improved risk ranking. After recalibration, O/E ratios generally returned toward 0.92 to 1.04, yet discrimination remained weak in multiple external cohorts (often *C*-statistics 0.50 to 0.60; and as low as 0.32 for recalibrated CCI in ELSA). This dissociation reinforces that calibration and discrimination are distinct properties: When the underlying linear predictor carries limited person-level risk gradient, intercept and slope updates can restore mean agreement but cannot add information [63].

To address transportability, we developed LLM-MMS as a bio-inspired AI multimorbidity score for human–machine clinical risk stratification rather than as a conventional diagnosis-counting score. Harmonized structured variables were converted into standardized English-language health narratives and processed with a prompt-constrained LLM framework to generate overall and organ-specific biological ages, frailty age, and multidimensional health grades; these outputs were then linked to a Cox model for all-cause mortality prediction. Conceptually, this pipeline abstracts several features of integrative clinical reasoning, including multimodal signal acquisition, cross-organ synthesis, estimation of biological vulnerability, and feedback of individualized risk. In this sense, LLM-MMS treats multimorbidity as a cross-domain phenotype that integrates diagnoses with functional, physiological, laboratory, behavioral, and social signals, while preserving clinician oversight through structured outputs and auditable interpretation. This human–machine framing is consistent with the broader view that medical AI should augment clinical decision-making by processing complex biomedical information at scale [64].

Across cohorts, LLM-MMS achieved higher and more consistent discrimination and markedly more stable calibration without cohort-specific retraining. Discrimination ranged from 0.67 in CLHLS (a very old, high-event-rate setting) to 0.86 to 0.87 in NHANES, remained strong in the UK Biobank internal validation (*C*-index 0.81), and reached 0.76 in CHARLS. External O/E ratios clustered tightly around 0.94 to 1.04. Subgroup analyses suggested mitigation of the “floor effect” seen with traditional indices in younger adults: Whereas conventional tools often lost discrimination below age 60 years, LLM-MMS retained useful separation in several cohorts, although sex-related heterogeneity remained evident in selected age-stratified subgroups. These sex-related differences may reflect cohort-specific variation in disease ascertainment, baseline risk, subgroup event numbers, behavioral and metabolic profiles, and the availability of sex-sensitive predictors.

Decision-curve analysis supported potential clinical relevance of LLM-MMS [53]. Across the external validation cohorts, LLM-MMS generally provided the highest or near-highest net benefit across threshold probabilities of 0.01 to 0.35, with average absolute net-benefit gains of approximately 0.035 to 0.065 and maximum gains of 0.043 to 0.130 compared with traditional indices. In Fig. 4, the *y*-axis was extended below zero because the treat-none strategy defines the reference net benefit of zero; truncating the axis at zero would obscure threshold ranges in which a model or default strategy yields negative net benefit. Negative net benefit indicates that, at those threshold probabilities, the harm-weighted burden of false-positive classification exceeds the benefit derived from true-positive classification. This finding suggests that, in the HRS 5-year setting, the available predictors may provide insufficient clinical utility for risk-guided classification across parts of the clinically plausible threshold range. In CLHLS, net-benefit curves were nearly overlapping across models, consistent with a potential information ceiling in a very old population with uniformly high short-term mortality risk; in such settings, alternative outcomes, such as disability, hospitalization, long-term care, or dynamic prediction, may be more informative than baseline mortality alone.

Because LLM-MMS depends on a foundation model at inference time, reproducibility and governance are central. We observed minimal performance variation across different base LLMs and negligible changes across repeated runs under fixed prompts (maximum Δ ≤0.004), supporting auditability. However, stability is not equivalent to safety: Widely deployed health algorithms can exhibit systematic and scalable bias [65]. Therefore, reporting aligned with the Transparent Reporting of a Multivariable Prediction Model for Individual Prognosis or Diagnosis statement [66] should be treated as minimum requirements, and model/prompt versioning with post-deployment monitoring should follow lifecycle principles for adaptive ML systems [67,68].

Computational cost is another practical consideration. The present implementation uses a 671-billion-parameter backbone, but inference was constrained to a single deterministic forward pass with greedy decoding, FP8-weight inference, and a single-call prompt architecture, rather than repeated sampling or multi-agent search. Moreover, the LLM is only required to generate the 17-dimensional phenotype; once generated, the Cox risk model is lightweight and can be deployed offline or through a web calculator. Thus, future implementation can use batched offline inference, scheduled phenotype updates, smaller validated backbones, or distilled surrogate models for routine screening.

Individuals in the LLM-MMS high-risk group exhibited marked proteomic abnormalities centered on inflammatory stress, epithelial dysfunction, tissue injury, and reduced physiological reserve. We observed significant up-regulation of GDF15, WFDC2, IL6, and KRT18. GDF15 is a stress-, infection-, and inflammation-induced cytokine that increases with aging and chronic inflammatory states [69]. Previous community-based cohort studies have shown that circulating GDF15 independently predicts all-cause, cardiovascular, and non-cardiovascular mortality, supporting its role as an integrative marker of systemic stress and reduced physiological reserve [70]. Consistently, large-scale plasma proteomics studies have identified GDF15 and WFDC2 among the strongest protein predictors of mortality across short- and long-term follow-up [71]. WFDC2, also known as HE4, is a secreted epithelial WAP-domain protein involved in epithelial host defense and innate immune responses in the respiratory and oral mucosa [72]. Elevated WFDC2/HE4 levels have also been associated with heart failure severity, impaired kidney function, and adverse clinical outcomes, suggesting that this protein may capture epithelial injury, fibrotic remodeling, and cardiorenal stress [73]. IL6 is a central pro-inflammatory cytokine and acute-phase mediator. Elevated circulating IL6 has been associated with mortality in older adults, and an inflammatory index incorporating IL6 and soluble tumor necrosis factor receptor-1 has been shown to predict 10-year all-cause mortality, supporting the interpretation that IL6 up-regulation reflects chronic systemic inflammatory burden [74,75]. KRT18 encodes keratin 18, an epithelial intermediate-filament protein. Circulating keratin 18 fragments are released during epithelial-cell apoptosis and necrosis and have been widely used as biomarkers of epithelial and hepatobiliary cell injury, indicating that KRT18 up-regulation in the high-risk group may reflect ongoing epithelial-cell damage and cell-death activity [76]. Therefore, the joint up-regulation of GDF15, IL6, KRT18, and WFDC2 supports an “inflammation–epithelial injury–systemic stress” coupling mechanism. Chronic inflammatory signaling and macrophage phenotype reprogramming represent key mediators linking disease burden to adverse clinical outcomes, as demonstrated in preclinical and oncology investigations [77,78]. In preclinical oncology work, extracellular-vesicle-mediated immune signal transduction has also been shown to shape inflammatory microenvironments [79]. Long-term multimorbidity burden may promote chronic inflammatory signaling, epithelial-cell injury and death, cardiorenal remodeling, and depletion of multi-organ functional reserve, ultimately contributing to increased mortality risk. Although this proteomic study was centered on a European population—which limits its generalizability—the LLM-driven framework has inherent scalability. Future studies should prioritize validation of the potential biological mechanisms underlying these findings. Multi-omics integration represents a powerful strategy for biomarker discovery, yet substantial practical implementation barriers have been well documented in oncology-oriented research settings [80,81]. Our approach avoids the cost and complexity of large-scale omics analyses, suggesting that clinical text mining may help identify important biomarkers for translational comorbidity research.

Explainability and reliability are essential for clinical use of medical AI [82]. We therefore evaluated LLM-MMS using complementary evidence from native reasoning traces, input-masking perturbation, and external epidemiological comparison. CoT prompting exposed intermediate reasoning traces for system-specific biological age and multidimensional health grading [31], and keyword summaries identified clinically plausible concepts across organ systems, including respiratory, cardiovascular, and metabolic domains (Figs. 6A and B). Beyond these descriptive traces, input-masking provided a more direct perturbation-based assessment [83]. Removing prespecified risk-factor information reduced the final LLM-MMS mortality risk score, with the largest effects for current smoking, inflammatory markers, systolic blood pressure, BMI, and HbA1c (Fig. 6C). Domain-specific masking showed coherent output changes, with smoking affecting behavioral and respiratory dimensions, inflammatory markers affecting immune and hematologic dimensions, systolic blood pressure affecting cardiovascular outputs, and BMI/HbA1c affecting metabolic-related outputs (Fig. 6D). The masking-derived ranking was also concordant with an external disability-adjusted-life-years-based ranking from the Global Burden of Disease Study 2021 [42] (Fig. 6E). Overall, these findings indicate that LLM-MMS can not only integrate multidimensional health information in multimorbidity risk assessment but also generate interpretable results consistent with clinical knowledge and population-level epidemiological evidence, providing a basis for its further use in multimorbidity identification, risk stratification, and clinical decision support.

Several limitations warrant emphasis. First, although LLM-MMS is framed as a “multimorbidity score”, it functions more like a prognostic health phenotype because it incorporates non-diagnostic domains (including socioeconomic measures). This broader scope improves mortality prediction but may be inappropriate for diagnosis-only risk adjustment and raises fairness and acceptability questions in payment or access-sensitive settings. Second, transportability might still be influenced by the UK Biobank training anchor, heterogeneous disease ascertainment across cohorts, and the textification step into English. Although the English reports were generated from harmonized structured variables rather than translated free-text records, we did not directly compare English-language prompts with Chinese-, Korean-, or Spanish-language prompts. Generalization to non-English workflows and to outcomes beyond all-cause mortality therefore requires direct evaluation. Finally, although hallucination risk was reduced through constrained inputs and templates, any narrative output should be treated as an auditable trace rather than clinical evidence.

In summary, traditional comorbidity indices showed large and unstable calibration drift on transport, and recalibration improved mean agreement but not risk ranking. LLM-MMS, without cohort-specific retraining, achieved higher discrimination, more stable calibration, and more consistent net benefit across cohorts and age–sex strata. These findings support LLMs as a methodological route to more transferable multimorbidity representations, provided that rigorous external validation, transparent governance, and fairness auditing accompany any real-world deployment.

## Data Availability

Data were obtained from the UK Biobank (UKB), the US National Health and Nutrition Examination Survey (NHANES), the Survey of Health, Ageing and Retirement in Europe (SHARE), the US Health and Retirement Study (HRS), the China Health and Retirement Longitudinal Study (CHARLS), the Chinese Longitudinal Healthy Longevity Survey (CLHLS), the English Longitudinal Study of Ageing (ELSA), the Korean Longitudinal Study of Aging (KLoSA), and the Mexican Health and Aging Study (MHAS). These datasets are hosted in publicly accessible research repositories and can be used by qualified researchers subject to each custodian’s registration, data-use terms, and (where applicable) application/approval procedures. This research was conducted using the UK Biobank Resource under Application Number 1150294; UKB data are available upon application to UKB (http://www.ukbiobank.ac.uk/) via the Access Management System (https://ams.ukbiobank.ac.uk/ams/). For other cohorts, data access is available after user registration (and any required agreements) through the respective official portals: NHANES (https://wwwn.cdc.gov/nchs/nhanes/), SHARE (https://share-eric.eu/data/become-a-user; https://share-eric.eu/data/data-access), HRS (https://hrsdata.isr.umich.edu/; https://hrsdata.isr.umich.edu/user/register), CHARLS (https://charls.pku.edu.cn/en/), CLHLS (https://opendata.pku.edu.cn/dataverse/CHADS), ELSA via the UK Data Service (Study 5050: https://datacatalogue.ukdataservice.ac.uk/studies/study/5050), KLoSA (https://survey.keis.or.kr/eng/klosa/klosa01.jsp), and MHAS (https://www.mhasweb.org/). Source data underlying the main results reported in this article are provided with this paper (Tables S17 to S34). The web-based survival prediction calculator used in this report is publicly available at http://llm-mms.xingyunjk.com/.
