# Supplementary material for: Bio-Inspired Artificial Intelligence Multimorbidity Score for Human–Machine Clinical Risk Stratification
Source: Cyborg Bionic Syst. 2026 Sep 30;7:0691. doi: 10.34133/cbsystems.0691 (PMC13624909; doi:10.34133/cbsystems.0691)
Supplement: Supplementary file 1 [file cbsystems.0691.f1.zip › Supplementary Appendix.docx]

**Supplementary appendix(method part)**

# Contents

**1. Supplementary Materials and Methods**

1.1	Ethics statement

1.2	LLM-MMI overview

1.3	Stage 1 — Data harmonisation and narrative construction

1.4	Stage 2 — Frozen policy backbone

1.5	Stage 3 — Hierarchical task decomposition

1.6	Stage 4 — Four-layer prompt schema

1.7	Stage 5 — Deterministic single-pass sampling

1.8	Stage 6 — Aggregation operator and Cox risk mapping

1.9	Cross-backbone portability hypothesis

1.10 Reproducibility hypothesis

1.11 Outcomes and predictor specification

1.12 Statistical analysis details

1.13 Biological correlates of LLM-MMI

1.14 Clinical interpretability of LLM-MMI

1.15 Implementation reference

1.16 Notation

2. Additional references introduced by this appendix

# 1. Supplementary Materials and Methods

## 1.1 Ethics statement

All participants included in the cohorts had provided written informed consent, and participation was voluntary with the right to withdraw at any time. The studies received ethical approval from their respective institutional review boards (IRBs) or ethics committees as follows: the UK Biobank was approved by the North West Multi-centre Research Ethics Committee, with data use governed by the relevant Material Transfer Agreement; the NHANES by the National Center for Health Statistics Research Ethics Review Board; and the HRS by the University of Michigan IRB. For the Chinese cohorts, the CHARLS received approval from the Peking University IRB (11015/11014), and the CLHLS from the Peking University Biomedical Ethics Committee (13074). European and other international cohorts were approved by the London Multi-Centre Research Ethics Review Committee (ELSA), the Max-Planck Society and University of Mannheim Ethics Council (SHARE), the Korea Centers for Disease Control and Prevention IRB (KLoSA), and the ethics bodies at the University of Texas Medical Branch, INEGI, and Mexico’s Instituto Nacional de Salud Pública (MHAS). Publicly available datasets (such as KLoSA and MHAS) and restricted datasets (UK Biobank) were utilized in strict accordance with their respective data usage policies.

## 1.2 LLM-MMI overview

LLM-MMI (Large-Language-Model-derived Multimorbidity Index) is a six-stage frozen-policy chain-of-thought reasoning pipeline that, in a single deterministic forward pass, transforms a participant’s harmonised structured record into a 17-dimensional multimorbidity representation suitable for Cox-based survival modelling. The six stages are: (1) deterministic structured-to-narrative harmonisation; (2) frozen-policy backbone configuration; (3) hierarchical task decomposition into a 18-node directed acyclic graph (DAG); (4) four-layer prompt schema (role, knowledge prior, typed chain-of-thought protocol, and JSON output contract); (5) deterministic single-pass greedy sampling; and (6) deterministic aggregation followed by a Cox proportional-hazards risk head with Level-2 recalibration on each external cohort. No parameters of the policy LLM are updated at any stage of the study; LLM-MMI therefore sits in the test-time-scaling family of large-language-model deployment described by Snell and colleagues.^80^

The architecture is a single-call, deterministic chain-of-thought (Wei *et al.*, NeurIPS 2022, Section 2)^31^, and is intentionally distinct from multi-call multi-agent reasoning frameworks such as ReAct^81^, AutoGen^82^, or process-reward-model search^83^: dependencies between sub-tasks are realised by topological ordering of fields inside one JSON object rather than by separate API calls. This design choice is motivated by population-scale inference (n = 770 439), by clinical auditability (a single deterministic artefact per participant), and by governance considerations for adaptive ML lifecycles described by the FDA and by PROBAST-AI.^19,67,68^

## 1.3 Stage 1 — Data harmonisation and narrative construction

Stage 1 is performed by a deterministic structured-to-narrative mapper *g* : *X* → V*, where *X* is the space of harmonised participant records and V* the space of English token sequences. Variables were harmonised using a unified medical dictionary that standardises definitions, units, coding, and time anchoring across five domains while retaining cohort provenance, so that each participant *i* is represented by

***x***_i_ = { x_i_^demo^, x_i_^life^, x_i_^exam^, x_i_^bio^, x_i_^hist^ },

corresponding to demographics, lifestyle, examinations, biomarkers, and medical history. The mapper produces *n*_i_ = *g*(***x***_i_) ∈ V*, an English natural-language report with standard units. To minimise hallucination risk, missing items are omitted rather than imputed with sentinel tokens; remaining variables are organised into hierarchical domain groups; and medical histories are rendered as chronologically normalised sub-narratives. Because *g* is fully decoupled from the language model, the same harmoniser is reused without modification across all nine cohorts and all evaluated backbones, isolating cohort heterogeneity to this single, auditable stage. The complete lists of indicators and fields used for harmonisation in each cohort (including Field ID, Indicator name, and domain Category) are provided in **Supplementary Tables 17–25** (one table per cohort). These tables collectively define the unified domain structure and variable inventory.

## 1.4 Stage 2 — Frozen policy backbone

Given context *c*, the policy LLM defines a token-level distribution

*πθ*(*y | c*) = ∏_t = 1 … |y|_ πθ(*y*_t_ | *c, y*_<t_),

shaped exclusively through the prompt template *P* and the decoding rule. No gradient updates, fine-tuning, or RLHF are applied at any point. Three considerations motivate this frozen-policy regime. First, population-scale inference makes any adaptive update on a held-out cohort cost-prohibitive. Second, fine-tuning on cohort C_j_ risks calibration drift on cohorts C_≠j_, a hazard echoed in the calibration-drift literature for clinical prediction models.^51,63^Third, clinical decision-support governance favours a versioned, frozen artefact, consistent with the FDA Predetermined Change-Control Plan guidance and the Good Machine Learning Practice principles.^19,67,68^

**Primary backbone.** Inference used DeepSeek-V3-0324, a 671-billion-parameter Mixture-of-Experts decoder-only transformer with 37 billion parameters activated per token (DeepSeek-AI *et al.*, arXiv:2412.19437; Architecture Section 2). The model employs Multi-head Latent Attention with low-rank key–value compression (Section 2.1.1) and DeepSeekMoE with auxiliary-loss-free load balancing across 256 routed experts plus a shared expert (Section 2.1.2), with eight active routed experts per token; a Multi-Token Prediction objective is used during pre-training (Section 2.2).^89,90^ The model was deployed on an HPC cluster of eight NVIDIA A100 (80 GB) GPUs with FP8 (E4M3) weights, weight-only FP8 quantisation (W8A16) on Ampere, and BF16/FP16 activations to reduce memory while preserving inference quality. The clinical reliability of DeepSeek-family models has been independently benchmarked in Sandmann *et al.*, Nature Medicine 2025.^89^

**Cross-backbone portability.** To assess whether LLM-MMI generalises beyond a single foundation model, the identical harmoniser, prompt template, and aggregation operator were reapplied unchanged on four further policies: GPT-5-nano (OpenAI),^33^ Grok-4-fast (xAI),^34^ Llama-3.3-70B-Instruct (Meta),^35,36^ and Gemini-2.5-flash-preview-05-20 (Google),^37^ with retrieval disabled in every backbone. Cross-backbone portability is defined formally as

*ℐ*_port_(π, π′) = | C-index(π) − C-index(π′) |,

with a prespecified portability hypothesis of max₊ *ℐ*_port_ < 0.02 with overlapping 95% CIs across the C(5,2) = 10 backbone pairs (Supplementary Table 6). The choice to evaluate five contemporaneous backbones spanning open-weight (Llama, DeepSeek), closed proprietary (GPT-5-nano, Gemini-2.5-flash), and hybrid (Grok-4-fast) ecosystems is intended to demonstrate that LLM-MMI’s representations are not idiosyncratic to a single training corpus or alignment stack.

## 1.5 Stage 3 — Hierarchical task decomposition

Multimorbidity assessment is decomposed into a directed acyclic graph *G* = (*T, E*) with 18 sub-task nodes:

*T* = { τ_0_ } ∪ *T*_organ_ ∪ *T*_ext_ ∪ { τ_17_ },

where τ_0_ performs multimorbidity pattern recognition and emits a rationale only; *T*_organ_ = { τ_1_, …, τ_10_ } collects nine organ-system biological ages (cardiovascular, digestive, respiratory, endocrine/metabolic, nervous, hematologic, musculoskeletal/motor, urinary, immune) following the heterogeneous-aging framework of Tian *et al.*, Nature Medicine 2023,^22^ together with a frailty age operationalised after Hanlon *et al.*, Lancet Public Health 2018^23^ and the Hospital Frailty Risk Score literature;^9^ *T*_ext_ = { τ_11_, …, τ_16_ } collects six external-factor grading dimensions (psychological health,^1,24^ diet,^25,26^ behaviours/habits,^27^ medical history,^1^ income,^1,28^ and family history^29^); and τ_17_ produces the integrative overall biological age, calibrated against the LLM-based biological-age framework reported by Li *et al.*, Nature Medicine 2025.^32^

The edge set

*E* = { τ_0_ → τ_k_ : 1 ≤ *k* ≤ 17 } ∪ { τ_k_ → τ_17_ : 1 ≤ *k* ≤ 16 }

enforces that the anchor node’s pattern recognition is shared context for every downstream sub-task and that the integrative sub-task depends on the union of upstream outputs, so that the same external exposure can yield different grades under different comorbidity profiles. Crucially, the DAG is unrolled inside a single prompt: dependencies are realised by the topological ordering of fields in the output JSON rather than by separate API calls. This makes LLM-MMI architecturally distinct from interactive multi-agent systems such as ReAct (Yao *et al.*, ICLR 2023),^81^ multi-call orchestration frameworks such as AutoGen (Wu *et al.*, arXiv:2308.08155),^82^ or process-reward-model-guided search (Lightman *et al.*, arXiv:2305.20050),^83^ all of which we explicitly do not adopt at population scale.

## 1.6 Stage 4 — Four-layer prompt schema

Each sub-task τ_k_ is realised by a virtual reasoning agent *A*_k_ = (ρ_k_, κ_k_, σ_k_), where ρ is a role specification, κ a domain-knowledge module, and σ an output schema. All agents share the same policy πθ and are instantiated jointly through a four-layer prompt template:

*P*(*n*_i_) = *R* ∥ *K* ∥ *S*_cot_ ∥ *S*_out_ ∥ *n*_i_,

**Role layer (R).** Declares a systems-medicine multimorbidity-expert persona that biases the policy toward weighting disease–disease interactions over additive effects, consistent with the multimorbidity epidemiology of Barnett *et al.*, Lancet 2012.^1^

**Knowledge layer (K).** Injects three priors. (i) A network-coupling prior over shared metabolic, inflammatory, endothelial/microcirculation, neurohumoral, and immune pathways, motivated by the disease-trajectory literature.^20^ (ii) A sentinel-indicator list of high-leverage markers (lactate, hypertensive crisis, GlycA, Cystatin C, RDW, low albumin, low vitamin D, Lp(a) with vascular leakage, neutrophils/NLR, GGT plus ketones, recurrent falls, insomnia, tooth loss, pulse pressure > 60 mmHg, ≥3 new diagnoses per year, monocytes > 10%, low urine creatinine, normal HbA1c with random glucose spikes, increased MPV, branched-chain amino-acid depletion, among others). (iii) An evidence-weighting rule under which objective laboratory indicators rank above subjective self-reports.

**Reasoning layer (S**_cot_**).** Scaffolds the chain of thought into three typed steps: (1) abnormal-value scan with weighting; (2) cross-system coupling analysis under the τ_0_ pattern; and (3) interaction-conditional grading—the same exposure must yield different grades under different comorbidity profiles. This typed protocol generalises the original chain-of-thought prompting method (Wei *et al.*, NeurIPS 2022, Section 2)^31^ by constraining the reasoning topology rather than relying on free-form intermediate steps.

**Output-contract layer (S**_out_**).** A strict JSON schema with seventeen (rationale, conclusion) fields in topologically sorted order. Numeric ages are integers in [25, 110]; gradings are drawn from { high-risk, medium-high-risk, medium-risk, medium-low-risk, low-risk }. The empirical schema-violation rate at temperature 0 was below 0.3% across all five backbones and all 770 439 inferences. The full prompt template, including the seventeen field definitions and the system-prompt header, is reproduced verbatim in Supplementary Table 2 and is used unchanged across cohorts and backbones; in particular, no cohort-specific tokens, language switches, or in-context examples are inserted.

## 1.7 Stage 5 — Deterministic single-pass sampling

For each sub-task τ_k_, agent *A*_k_ generates a rationale–conclusion pair

(*r*_i_^(k)^, *z*_i_^(k)^) ∼ πθ( · | *P*(*n*_i_), τ_k_),

with greedy decoding *y*_t_ = arg max_v ∈ V_ πθ(*v | c, y*_<t_). Stochastic test-time-scaling strategies—majority voting, best-of-N sampling, beam search with process-reward models,^83^ or Monte Carlo tree search—are not used, for two reasons. First, population-scale inference (n = 770 439) makes multi-sample strategies cost-prohibitive even at moderate sample counts. Second, clinical auditability favours a deterministic pipeline, so that re-running the same input under the same software stack produces a bit-identical output that a regulator or reviewer can re-derive. The empirical reproducibility of this choice is quantified by

*Δ*_repro_ = max_1 ≤ m, m′ ≤ 5_ | C_m_ − C_m′_ |,

where C_m_ is the Harrell’s C-index of the m-th independent run on a held-out cohort under identical inputs and prompts. We observed Δ_repro_ ≤ 0.004 on NHANES across five independent runs of the primary backbone (Supplementary Table 7), consistent with the bit-identical behaviour expected from greedy decoding under a fixed model and the corresponding inference precision regime described by DeepSeek-AI.^90^

## 1.8 Stage 6 — Aggregation operator and Cox risk mapping

Let σ_ord_ map ordinal grades to integers (low-risk = 1, medium-low-risk = 2, medium-risk = 3, medium-high-risk = 4, high-risk = 5). The aggregation operator Φ is the deterministic identity stack

***z***_i_ = Φ( { *z*_i_^(k)^ }_k = 1 … 17_ ) = [ *z*_i_^(1)^, …, *z*_i_^(17)^ ]ᵀ ∈ ℝ¹⁷.

This linear stack is intentionally trivial: all non-linearity, prior knowledge, and disease–disease interactions live inside πθ, while the head remains transparent and TRIPOD-/PROBAST-AI-compatible (Collins *et al.*, BMJ 2015;^66^ Moons *et al.*, BMJ 2025^19^). The Cox proportional-hazards model

*h*(*t |* ***z***_i_) = *h*_0_(*t*) · exp( βᵀ ***z***_i_ )

is fitted on the UKB development cohort by partial-likelihood maximisation:

β̂ = arg max₊ ∑_i : δi = 1_ [ βᵀ ***z***_i_ − log ∑_j ∈ R(ti)_ exp( βᵀ ***z***_j_ ) ],

with R(*t*) the risk set at time *t* and δ_i_ the event indicator. The use of risk-period waves is supported by the counting-process formulation of Andersen and Gill, Annals of Statistics 1982,^21^ so that within-participant repeated assessments contribute additional risk intervals without double-counting events.

**Level-2 recalibration.** For each external cohort C_j_, we apply intercept-and-slope (Level-2) recalibration^51^ by refitting

Surv(*t, δ*) ∼ α + γ · lpθ	on	C_j_,

where lpθ,*i* = β̂ᵀ ***z***_i_ is the linear predictor from the development model. The recalibrated absolute *t*-year risk for participant *i* ∈ C_j_ is

*p̂*_i_(*t*) = 1 − exp[ − H_0_^recal^(*t*) · exp( α̂ + γ̂ · lpθ,*i* ) ],

with H₀^recal^(*t*) the Breslow estimator from the recalibration fit. This is the recalibration policy recommended by Van Calster *et al.*, J Clin Epidemiol 2016, Levels 1–4 hierarchy,^51^ and accommodates the calibration-drift phenomenon emphasised by Van Calster *et al.*, BMC Medicine 2019.^63^ The web-based survival-prediction calculator implementing the final workflow is publicly available at http://llm-mmi.xingyunjk.com/.

## 1.9 Cross-backbone portability hypothesis

To establish that LLM-MMI is not dependent on a single foundation model, we prespecified the portability hypothesis max₊ *ℐ*_port_(π, π′) < 0.02 across the C(5,2) = 10 ordered pairs of backbones (DeepSeek-V3-0324, GPT-5-nano, Grok-4-fast, Llama-3.3-70B-Instruct, Gemini-2.5-flash-preview-05-20), evaluated on the NHANES external cohort with overlapping 95% CIs. The harmoniser *g*, prompt template *P*, DAG *G*, aggregation operator Φ, and Cox head are held identical across backbones, so any residual difference in C-index is attributable to the policy alone (Supplementary Table 6). The cross-backbone evaluation also tests whether contextual prior knowledge accumulated by larger or differently-aligned LLMs translates into a downstream survival-prediction advantage; under our deterministic single-pass protocol, that question reduces to whether different policies produce systematically different ***z***_i_ vectors for the same narrative. The five-backbone panel additionally serves as a rough robustness check against backbone-specific failure modes (e.g., refusal patterns, formatting drift, or systematic numerical bias).

## 1.10 Reproducibility hypothesis

Even with greedy decoding, two distinct sources of stochasticity can perturb LLM outputs at population scale: floating-point non-determinism in MoE routing and kernel-level non-determinism in the inference engine. To bound their joint effect, we prespecified the reproducibility hypothesis Δ_repro_ ≤ 0.005 (Harrell’s C) on NHANES across five independent runs of the primary backbone under identical inputs and prompts. The observed value was Δ_repro_ ≤ 0.004, which together with the cost considerations above justifies single-pass deterministic chain-of-thought relative to majority-voting or best-of-*N* alternatives at this scale (Supplementary Table 7).

## 1.11 Outcomes and predictor specification

The primary outcome was all-cause mortality. Follow-up was measured in days from baseline (or wave-specific index date under the risk-period framework) to death or censoring. The predictor vector ***z***_i_ ∈ ℝ¹⁷ comprised the seventeen LLM-MMI dimensions: (i) cardiovascular system age; (ii) digestive system age; (iii) respiratory system age; (iv) endocrine/metabolic system age; (v) nervous system age; (vi) hematologic system age; (vii) musculoskeletal/motor system age; (viii) urinary system age; (ix) immune system age; (x) frailty age; (xi) psychological health grading; (xii) dietary health grading; (xiii) behavioural/habit health grading; (xiv) medical history grading; (xv) income grading; (xvi) family history grading; and (xvii) overall biological age. Benchmark indices were Quan’s Charlson index (Quan *et al.*, Am J Epidemiol 2011),^5^ the functional comorbidity index (Sears and Rundell, Med Care 2020),^38^ the modified hospital frailty risk score (Sim *et al.*, J Frailty Sarcopenia Falls 2024),^39^ the Elixhauser–van Walraven score (van Walraven *et al.*, Med Care 2009),^7^ and a multimorbidity-weighted index (Wei *et al.*, BMJ Open 2024),^40^ each used at its published definition.

## 1.12 Statistical analysis details

**Validation strategy.** Validation followed TRIPOD-aligned reporting (Collins *et al.*, BMJ 2015, Items 10–20 of the checklist),^66^ and PROBAST-AI quality, risk-of-bias, and applicability assessment (Moons *et al.*, BMJ 2025).^19^ Internal validation in UKB used 200 bootstrap resamples for optimism-corrected discrimination metrics following Steyerberg *et al.*, J Clin Epidemiol 2001–2003,^47,48^ and Harrell *et al.*, Stat Med 1996.^49^ External validation used the remaining eight cohorts, with Level-2 recalibration to address baseline-risk heterogeneity (Van Calster *et al.*, J Clin Epidemiol 2016, Levels 1–4).^51^

**Discrimination.** Reported as Harrell’s C-index (Harrell *et al.*, Stat Med 1996, Section 3)^49^ with asymptotic 95% CIs derived from the variance estimator of the concordance statistic (Uno *et al.*, Stat Med 2011, Section 2);^55^ a sensitivity analysis used Uno’s truncation-aware C-statistic from the same paper (Section 4).

**Calibration.** Five-year and ten-year calibration was assessed with calibration plots stratified by deciles of predicted risk, calibration slopes, and observed-to-expected (O/E) ratios; 95% CIs for the O/E ratio used the log-Poisson method based on the observed event count.

**Prediction error.** Ten-year prediction error used the inverse-probability-of-censoring-weighted (IPCW) Brier score (Graf *et al.*, Stat Med 1999, building on the original probabilistic-forecast verification framework of Brier 1950)^52^, together with a Brier-based R² of the form 1 − Brier_model_ / Brier_null_.

**Decision-curve analysis.** Survival-adapted net benefit with censoring adjustment over threshold probabilities of 0.01–0.35 (Vickers and Elkin, Med Decis Making 2006, with the survival extension of Vickers *et al.*, BMC Med Inform Decis Mak 2008).^53,54^

**Assumptions and subgroups.** The proportional-hazards assumption was assessed graphically via scaled Schoenfeld residuals. Subgroup analyses were prespecified by sex (female/male), age (<60 / ≥60 years), and the four sex × age strata. Analyses used R 4.3.3 (*survival, dcurves, ggplot2*) and Python 3.10.8; two-sided *p* < 0.05 defined statistical significance. The implementation reference for the stratification loop and asymptotic CI helpers is given in Section 1.16.

## 1.13 Biological correlates of LLM-MMI

To explore the biological correlates and downstream prognostic utility of LLM-MMI, we conducted complementary proteomic analyses in the proteomics-enrolled subset of UKB. We compared protein-abundance profiles between participants with high and low multimorbidity burden as defined by the prespecified burden stratification used in the main analysis. Differential abundance was assessed using age- and sex-adjusted linear models, and *p* values were corrected for multiple testing using the Benjamini–Hochberg procedure. Proteins meeting the predefined statistical-significance and effect-size criteria were regarded as candidate biomarkers of high comorbidity burden and were visualised with volcano plots. To characterise the biological processes represented by these proteins, we performed pathway enrichment on the ranked differential-expression results and summarised the significantly enriched pathways using enrichment-score plots, motivated by the high-coverage proteomic-mortality framework reported by Eiriksdottir *et al.*, Communications Biology 2021,^71^ and the proteomic-risk-prediction analysis of Carrasco-Zanini *et al.*, Nature Medicine 2024.^91^

We then evaluated the prognostic relevance of candidate proteins for all-cause mortality by fitting Cox proportional-hazards models in the proteomic subset and reporting the C-index with 95% CIs for each protein. Finally, to assess whether multimorbidity-related protein signatures could improve risk stratification, we trained an XGBoost-Cox model on the selected candidate proteins and quantified predictive performance with time-dependent ROC curves and corresponding AUC values at 1, 3, 5, 10, and 15 years.

## 1.14 Clinical interpretability of LLM-MMI

Clinical interpretability of LLM-MMI was operationalised at three complementary levels: native-trace interpretation, post-hoc local attribution, and global surrogate modelling.

**Native chain-of-thought traces.** Per-task rationales emitted under the four-layer prompt schema (Section 1.6) were post-processed using standard text normalisation (lowercasing, lemmatisation, stop-word removal) and keyword extraction. Frequent and informative terms were summarised as word clouds for the overall biological age, organ-specific biological ages, frailty age, and the six external-factor gradings. This native-trace channel exists because the policy LLM is deterministic under greedy decoding (Section 1.7) and is required by the prompt to emit a rationale field before each conclusion field, so that every numerical output is paired with its own auditable reasoning.

**1.15 Implementation reference**

The complete inference pipeline is implemented as the llm_mmi/ Python package; statistical analysis is implemented in eval/ukb.R (univariate baselines) and eval/other.R (multivariate LLM-MMI). The mapping is one-to-one with the methodology stages in Sections 1.4–1.8.

| **Stage** | **Methods text anchor** | **Code location** | **Entry point / knob** |
| --- | --- | --- | --- |
| 1. g(·) | Stage 1 — Data harmonisation and narrative construction | External preprocessing | input column of the cohort Excel files |
| 2. πθ | Stage 2 — Frozen policy backbone | llm_mmi/config.py | POLICY_BACKBONES; env var LLM_MMI_BACKBONE |
| 3. G | Stage 3 — Hierarchical task decomposition | llm_mmi/schema.py | render_json_schema(decomposition_k, anchor_disease_pattern, per_task_cot) |
| 4. P | Stage 4 — Four-layer prompt schema | llm_mmi/prompt.py | assemble_prompt(narrative, ablation) |
| 5. greedy decoding | Stage 5 — Deterministic single-pass sampling | llm_mmi/inference.py | _process_row() |
| 6. Φ + Cox + Level-2 | Stage 6 — Aggregation and Cox risk mapping | eval/other.R | run_one_analysis_single() |

## 1.16 Notation

The notation used throughout this appendix is collected below for convenience.

| **Symbol** | **Meaning** |
| --- | --- |
| x_i ∈ X | Participant i’s harmonised record over five domains (demographics, lifestyle, examinations, biomarkers, medical history). |
| g(·) | Deterministic structured-to-narrative harmoniser; n_i = g(x_i). |
| n_i ∈ V* | English health narrative (token sequence) for participant i. |
| πθ | Frozen policy LLM; parameters θ are unchanged at every stage of the study. |
| T = { τ_0, …, τ_17 } | 18-node directed-acyclic decomposition of the multimorbidity assessment task. |
| τ_0 | Anchor sub-task (multimorbidity pattern recognition; rationale-only). |
| T_organ = { τ_1, …, τ_10 } | Nine organ-system biological ages plus frailty age. |
| T_ext = { τ_11, …, τ_16 } | Six external-factor grading dimensions. |
| τ_17 | Integrative overall biological age. |
| A_k = (ρ_k, κ_k, σ_k) | Virtual reasoning agent for sub-task τ_k. |
| P = R ∥ K ∥ S_cot ∥ S_out ∥ n | Concatenated four-layer prompt template. |
| r_i^(k), z_i^(k) | Rationale and conclusion of agent k on participant i. |
| z_i ∈ ℝ¹⁷ | Aggregated multimorbidity representation. |
| Φ | Aggregation operator (deterministic identity stack). |
| h(t \| z_i) = h_0(t) exp(βᵀ z_i) | Cox proportional-hazards model for all-cause mortality. |
| lp_θ,i = β̂ᵀ z_i | Linear predictor under the development model. |
| α̂, γ̂ | Level-2 recalibration intercept and slope (per external cohort). |
| H_0^recal(t) | Breslow baseline hazard from the recalibration fit. |
| ℐ_port(π, π′) | Cross-backbone portability metric. |
| Δ_repro | Five-run reproducibility metric on the held-out cohort. |

# 2. Additional references introduced by this appendix

The references below are introduced relative to the main-text reference list (numbered 1–79). They support the reasoning-LLM positioning of LLM-MMI and are cited above by their reference number. Where relevant, the section or page within the cited paper is given to facilitate verification.

**[80]**	Snell C, Lee J, Xu K, Kumar A. Scaling LLM test-time compute optimally can be more effective than scaling model parameters. arXiv:2408.03314 (2024). Cited in Section 1.2 to position LLM-MMI within the test-time-scaling spectrum (Section 3 “Scaling test-time compute”).

**[81]**	Yao S, Zhao J, Yu D, Du N, Shafran I, Narasimhan K, Cao Y. ReAct: synergising reasoning and acting in language models. ICLR (2023); arXiv:2210.03629. Cited in Section 1.2 and Section 1.5 to contrast LLM-MMI’s single-call deterministic chain-of-thought with interactive multi-step reasoning that interleaves “thought” and “action” tokens (Section 2).

**[82]**	Wu Q, Bansal G, Zhang J, et al. AutoGen: enabling next-generation LLM applications via multi-agent conversation. arXiv:2308.08155 (2023). Cited in Section 1.5 as the multi-call multi-agent comparator we explicitly do not adopt at population scale (Section 3 “Agent design”).

**[83]**	Lightman H, Kosaraju V, Burda Y, et al. Let’s verify step by step. arXiv:2305.20050 (2023). Cited in Section 1.5 and Section 1.7 as the process-reward-model reference we explicitly do not adopt for stochastic test-time-scaling search (Section 4 “Process- vs outcome-supervised reward models”).

**[84]**	Vaswani A, Shazeer N, Parmar N, et al. Attention is all you need. NeurIPS (2017); arXiv:1706.03762. Cited in Section 1.4 as the architectural foundation underlying all five evaluated decoder-only backbones (Section 3 “Model architecture”).

**[85]**	Ouyang L, Wu J, Jiang X, et al. Training language models to follow instructions with human feedback (InstructGPT). NeurIPS (2022); arXiv:2203.02155. Cited in Section 1.4 to motivate why the alignment stack of each evaluated backbone may differ while leaving downstream LLM-MMI performance comparable (Section 3 “Methods”).

**[86]**	Graf E, Schmoor C, Sauerbrei W, Schumacher M. Assessment and comparison of prognostic classification schemes for survival data. Stat Med 1999; 18: 2529–45. Cited in Section 1.12 as the IPCW Brier-score reference (Section 3 “Time-dependent Brier score”).

**[87]**	Cox DR. Regression models and life-tables. J R Stat Soc B 1972; 34: 187–220. Cited in Section 1.8 as the original Cox proportional-hazards model (Equations 1–3).

**[88]**	Wolff RF, Moons KGM, Riley RD, et al. PROBAST: a tool to assess the risk of bias and applicability of prediction model studies. Ann Intern Med 2019; 170: 51–58. Cited in Section 1.12 as the predecessor of PROBAST-AI used in our risk-of-bias appraisal (Box 2 of the original paper).

**[89]** Sandmann S, et al. Benchmark evaluation of DeepSeek large language models in clinical decision-making. Nat Med 2025; 31: 2546–2549. Cited in Section 1.4 to support the statement that the clinical reliability of DeepSeek-family models has been independently benchmarked.

**[90]** DeepSeek-AI, et al. DeepSeek-V3 Technical Report. arXiv:2412.19437 (2025). Cited in Section 1.4 to document the DeepSeek-V3-0324 architecture, including Multi-head Latent Attention, DeepSeekMoE, routed experts, auxiliary-loss-free load balancing, and multi-token prediction; also cited in Section 1.7 for the corresponding inference-precision regime.

**[91]** Carrasco-Zanini J, et al. Proteomic signatures improve risk prediction for common and rare diseases. Nat Med 2024; 30: 2489–2498. Cited in Section 1.13 as the proteomic-risk-prediction reference motivating the downstream proteomic analyses.
