# Supplementary material for: Bio-Inspired Artificial Intelligence Multimorbidity Score for Human–Machine Clinical Risk Stratification
Source: Cyborg Bionic Syst. 2026 Sep 30;7:0691. doi: 10.34133/cbsystems.0691 (PMC13624909; doi:10.34133/cbsystems.0691)
Supplement: Supplementary file 1 [file cbsystems.0691.f1.zip › Supplementary Table 1.docx]

**Supplementary Table 1. Data Availability and Characteristics of Selected Risk Factors in Nine Included Cohorts**

| **Predictor** | **NHANES** | **UKB** | **MHAS** | **KL** | **SHARE** | **ELSA** | **HRS** | **CLHLS** | **CHARLS** |
| --- | --- | --- | --- | --- | --- | --- | --- | --- | --- |
| **Age** | ✔^a^ | ✔ | ✔ | ✔ | ✔ | ✔ | ✔ | ✔ | ✔ |
| **Sex** | ✔ | ✔ | ✔ | ✔ | ✔ | ✔ | ✔ | ✔ | ✔ |
| **Laboratory test** | ✔ | ✔ | ✔ |  |  | ✔ |  |  |  |
| **Physical examination** | ✔ | ✔ | ✔ | ✔ | ✔ | ✔ | ✔ | ✔ | ✔ |
| **Blood analysis** | ✔ | ✔ |  |  |  | ✔ |  |  |  |
| **Questionnaire** | ✔ | ✔ | ✔ | ✔ | ✔ | ✔ | ✔ | ✔ | ✔ |

Note: ^a^ ✔ indicates that the data for the specific risk factor or category are available in the respective cohort.

Abbreviations: CHARLS, China Health and Retirement Longitudinal Study; CLHLS, Chinese Longitudinal Healthy Longevity Survey; ELSA, English Longitudinal Study of Ageing; HRS, Health and Retirement Study; KL, Korean Longitudinal Study of Ageing (KLoSA); MHAS, Mexican Health and Aging Study; NHANES, National Health and Nutrition Examination Survey; SHARE, Survey of Health, Ageing and Retirement in Europe; UKB, UK Biobank.
